# Leveraging machine learning for digital gait analysis in ataxia using sensor-free motion capture

**DOI:** 10.1038/s43856-025-01258-y

**Published:** 2026-01-27

**Authors:** Philipp Wegner, Marcus Grobe-Einsler, Lara Reimer, Fabian Kahl, Berkan Koyak, Tim Elter, Alexander Lange, Okka Kimmich, Daniel Soub, Felix Hufschmidt, Sarah Bernsen, Mónica Ferreira, Thomas Klockgether, Jennifer Faber

**Affiliations:** 1 German Center for Neurodegenerative Diseases (DZNE), Bonn, Germany; 2 University of Bonn, Bonn, Germany; 3 Center for Neurology, Deptment of Parkinson’s Disease, Sleep and Movement Disorders, University Hospital Bonn, Bonn, Germany; 4 Institute for Digital Medicine, University Hospital Bonn, Bonn, Germany; 5 Center for Neurology, Deptment of Vascular Neurology, University Hospital Bonn, Bonn, Germany; 6 Center for Neurology, Deptment of Neuromuscular Diseases, University Hospital Bonn, Bonn, Germany; 7 Deptment of Neuroradiology, University Hospital Bonn, Bonn, Germany

**Keywords:** Movement disorders, Predictive markers, Neurodegenerative diseases, Computational neuroscience

## Abstract

**Background:**

Gait disturbances are the clinical hallmark of ataxia. Their severity is assessed within a well-established clinical scale, which only allows coarse scoring and does not reflect the complexity of individual gait deterioration. We investigated whether sensor-free motion capture enables to replicate clinical scoring and improve the assessment of gait disturbances.

**Methods:**

The normal walking task during clinical assessment was videotaped in 91 ataxia patients and 28 healthy controls. A full-body pose estimation model (AlphaPose) was used to extract positions, distances, and angles over time while walking. The resulting time series were analyzed with four machine learning (ML) models, which were combinations of feature extraction (tsfresh, ROCKET) and prediction methods (XGBoost, Ridge). First, in a regression and classification approach, we trained the ML models on reconstructing the clinical score. Second, we used explainable AI (SHAP) to identify the most important time series. Third, we investigated time series features to study longitudinal changes.

**Results:**

Gait disturbances are assessed with high accuracy by ML models, slightly improving human rating (i) in the categorial prediction of the clinical score (F1-score best model: 63.99%, human: 60.57% F1-score), (ii) in the detection of subtle changes (pre-symptomatic patients, clinically rated unimpaired are differentiated from HC with a F1-score of 75.96%) and (iii) in the detection of longitudinal changes over time (Pearson’s correlation coefficient model: −0.626, *p* < 0.01; human: −0.060, not significant).

**Conclusions:**

ML-based analysis shows improved sensitivity in assessing gait disturbances in ataxia. Subtle and longitudinal changes can be captured within this study. These findings suggest that such approaches may hold promise as potential outcome parameters for early interventions, therapy monitoring, and home-based assessments.

## Plain language summary

This study explored a way to measure walking problems in people with ataxia, a condition that affects balance and movement. Researchers used video recordings of patients and healthy participants while walking and analyzed them with a machine learning model that tracks body movements without needing sensors. The model was used to predict clinical scores of walking difficulties and to detect subtle changes over time. The results showed that this approach can capture walking problems accurately and may help detect early changes before symptoms appear, as well as track changes over time. This method could support earlier interventions, improved therapy monitoring, and even enable home-based assessments for people with ataxia.

## Abstract (web summary)

Wegner et al. apply machine learning to video recordings of walking tasks in people with ataxia and healthy controls using sensor-free motion capture. Their models replicate clinical ratings and detect subtle longitudinal gait changes that are challenging to capture in standard assessments, supporting improved monitoring of disease progression.

## Introduction

Neurodegenerative ataxias are a group of sporadic and hereditary movement disorders characterized by a progressive loss of balance and coordination accompanied by slurred speech, leading to increasing disability and premature death. Gait disturbances are one of the main symptoms in ataxias resulting in substantial restriction of mobility with the need to use walking aids and, finally, the loss of ambulation in later stages of the disease. Initially, imbalance and resulting gait disturbances become obvious only during challenging tasks, such as a tandem walk or on uneven ground. As the disease progresses, the normal gait becomes increasingly impaired as well. Since gait disturbances are one of the core clinical hallmarks of ataxias, the clinical onset of the disease is most often defined as the patient’s reported onset of gait disturbances^1–3^. The clinical scale used for the assessment and rating of ataxia includes gait as the first item (Scale for the Assessment and Rating of Ataxia, SARA)^4^. Here, a person’s gait is rated from 0 (normal) to 8 (unable to walk) based on two tasks: The person is asked (1) to walk at a safe distance parallel to a wall including a half-turn (turn around to face the opposite direction of gait) and (2) to walk in tandem (heels to toes) without support. The clinical rating is based on parameters such as missteps in tandem walk or staggering. The tandem walk task is mandatory for the discrimination between SARA gait scores 0 and 1. An unimpaired, normal gait with no difficulties in walking and turning, as well as an unimpaired tandem walk, is rated with 0, while the combination of an unimpaired normal gait but “*slight difficulties, only visible when walking 10 consecutive steps in tandem*” is rated with 1^4^. For ataxia patients, alterations in, for instance, step width, decreased step length, and increased variability in foot placement and general trajectories have been described^5–7^. These abnormalities could be assessed using wearable sensors^8–10^. Sensor-free motion capture has been used to study gait disturbances in a rodent model of ataxia^11^. Lang et al. were able to characterize ataxia-specific movement and further quantify subtle movement changes that could not be identified visually, utilizing a single-camera setup in combination with a 2D pose estimation framework. Digital gait assessments have been studied in ataxias and other movement disorders, showing promising results even in the context of therapeutic interventions. Prior efforts have used multi-camera setups in combination with force plates and a Kinect v2 sensor^12^, single-camera with deep learning-based 2D pose estimation^13,14^, single-camera motion capturing using optical markers in combination with inertia sensors^15^, infrared depth sensors^16^. Moreover, efforts have been made to compare these technologies in the context of clinical applications^17^. This related work provides a strong foundation for digital motion analysis in ataxia disorders. However, the type of analysis and methodologies differ from our approach by either focusing on other movement disorders, such as Parkinson’s, using a different technology, like sensors or wearable devices, introducing new measures (e.g., Pose Dispersion Index), or investigating an entirely different species, such as rodents. To the best of our knowledge, no study has been published to date that aims to reconstruct a SARA item solely from video data in a mixed ataxia cohort of this size. This gap in the literature served as the motivation for this work and highlights its contribution to advancing digital gait analysis in ataxia.

In this work, we aim to characterize gait disturbances in an adult cohort of 91 ataxia patients suffering from sporadic neurodegenerative or hereditary ataxias, as well as healthy controls (HC), autonomously by utilizing multiple machine learning models within a straightforward sensor-free setting. For this purpose, we use participants’ normal gait that is videotaped during clinical examinations (*N* = 159). First, a deep learning-based sensor-free motion capture model is used to quantify a person’s gait by extracting time series of body markers and subsequently characterizing features thereof. Second, machine learning models are trained to reproduce the clinical classification, employing the human examiner’s SARA gait item scoring as ground truth. Third, we conduct a feature importance investigation to identify those features, that are most important for the final model prediction. Fourth, we assess the sensitivity of key digital parameters to longitudinal change and compare their trajectories with those of the clinical scale. This study investigates how machine learning can (i) reconstruct a clinical rating score determined by a trained neurologist, and (ii) improve sensitivity in detecting subtle and longitudinal changes. Especially in those hereditary ataxias for which gene therapies are currently being tested in phase 1 trials (clinicaltrials.gov NCT05822908), the detection of early, subtle gait pathologies is of particular interest. Given safety and tolerability, such interventions would, in principle, offer the intriguing option of preventive trials. Thus, digital biomarkers that allow to capture subtle, early changes, would support the planning of future preventive trials. The approach presented in this work offers great opportunities to assess ataxia diseases more fine-grained and personalized, which ultimately allows improved disease modeling. Accurate modeling of the disease is crucial for the success of any clinical trial investigating the effectiveness of a medication, especially in ataxias, which are typically slowly progressing^18^. Moreover, the video-based assessment of gait disturbances based on sensor-free motion capture is beneficial as it can be easily integrated into the clinical routine and could even be performed at home, allowing closer monitoring of treatment responses and daily fluctuations. Note that in this work, we use the term ’sensor-free’ to emphasize the absence of wearable devices, and ’video-based’ to indicate that motion capture and gait analysis are performed using videotaped assessments. While similar approaches are often described in the literature as markerless motion capture, we use sensor-free motion capture to highlight that our method relies solely on video data, without any wearable sensors. This distinguishes our approach from other commonly used gait analysis methods, such as sensor-based motion capture or systems using inertial or pressure sensors^8,9^, which do require specialized hardware. This study shows that sensor-free motion capture on videotaped clinical assessments, in combination with a suitable machine learning model, is capable of accurately reconstructing clinical ratings that are currently assigned by a trained professional onsite. The presented methodology slightly improves on the human baseline performance (*F*_1_-score best model: 63.99%, human: 60.57% *F*_1_-score), is capable of distinguishing healthy controls from pre-symptomatic patients (*F*_1_-score of 75.96%), and models gait disturbances longitudinally more accurately than the clinical score itself (Pearson’s correlation coefficient model: −0.626, p < 0.01; human: −0.060, not significant). Subsequent explainable AI investigations reveal that upper limb movement is most important for the model to distinguish between different SARA gait scores. Finally, the presented work has potential in multiple aspects of ataxia research and care. Firstly, this study shows that machine learning applied to sensor-free video recordings of walking can detect subtle and early gait disturbances in ataxia, including changes not visible to human examiners. This potentially allows for earlier intervention and more accurate disease monitoring. Secondly, the method is practical for clinical and home use, which enables performing ataxia assessments on a large scale either at home or in a clinical setting without the necessity for a SARA-trained professional.

## Methods

### Data

#### Participants and clinical assessment

All participants were enrolled in ongoing observational studies in neurodegenerative ataxias at the German Center for Neurodegenerative Diseases (Deutsches Zentrum für Neurodegenerative Erkrankungen, DZNE) in Bonn, Germany, and a full list of studied patient subgroups of the DZNE Clinical Ataxia Network (DCAN) is given in Supplementary Table 1. Of the in total 119 subjects included, 28 were healthy controls and 91 were ataxia patients of the following distribution: 58 spinocerebellar ataxia (SCA)(SCA3: 28, SCA6: 10, SCA1: 10, SCA2: 4, SCA10: 2, SCA8: 1, SCA5: 1, SCA26: 1, not further specified SCA: 1), 2 early-onset ataxias of unknown etiology, 11 multiple system atrophy of cerebellar type (MSA-C) as well as 6 sporadic adult-onset ataxia of unknown etiology (SAOA)), 3 FXTAS, 2 BRAT1, 2 SYNE1, 2 sporadic ataxias suspect for autoimmune ataxia, 2 Friedreich-Ataxia, 1 CTX, 1 RFC1 and 1 CANCA1A missense mutation. The final cohort was found to be sufficient in size for the analysis carried out in this work. All participants underwent a standardized clinical assessment including the Assessment and Rating of Ataxia (SARA)^4^ and the majority additionally completed the Inventory of Non-Ataxia Signs (INAS)^19^, between October 2018 and December 2023. SARA includes assessment and rating of 8 different items. The first item, gait, includes the assessment of a normal walking task, which was used for the automated video analyses (see next paragraph for more details), and a tandem walk. Instruction and graded rating scores for the SARA item gait are provided in Supplementary Fig. 1. Notably, the differentiation between grade 0 (normal) and 1 refers to gait disturbances, which are only obvious during the tandem walk task, while the normal gait is unimpaired according to the examiner. The study was approved by the local ethics committee (Ethics Committee of the Medical Faculty of the University of Bonn, https://ethik.meb.uni-bonn.de/), and all participants gave their written consent, including the assessments and the videotaping, according to the Declaration of Helsinki.

#### Video taping for sensor-free motion capture and selection of videos

During visits, the regular SARA assessments were videotaped following a standardized protocol. For the gait item, the camera was positioned directly in front of the person (Supplementary Fig. 2). Notably, for the subsequent motion capture analysis, we only used the videos of task 1 of the SARA gait item, the gait task of normal walking. For quality control, all videos were inspected visually for suitability and excluded otherwise. Reasons for exclusion were: disturbances of the overall scene by other persons (e.g., clinic personnel), insufficient video quality, or environmental factors, such as mirroring surfaces, that disrupted the downstream data processing, for instance, the motion capture. We restricted the analysis to those patients who could perform the normal gait task without walking aids, which corresponds to a SARA gait score of less than 5. All videos were recorded using an iPad Pro (2nd generation) with a frame rate of 30 at a resolution of 1920×1080. In the end, each video was labeled with its respective on-site SARA gait score and additionally tagged as HC or ataxia patient.

### Human rating

Human examiner rating of the SARA gait item is based on both tasks, normal gait, and tandem walk. The on-site assessment of the SARA gait item was conducted and rated by experienced neurologists who were trained to perform the SARA. This on-site rating was considered as ground truth for the SARA gait score. A subset of video recordings of the SARA gait item, again including both tasks (*N* = 41), was rated *a posteriori* by three clinical experts in consensus as part of the SARA training tool development^20^. These consensus video ratings were considered a human prediction and likewise compared with the ground truth of the on-site ratings. The resulting performance scores were taken as baseline performance for this work.

### Motion capture

For motion capture, we used Alpha Pose, a full-body pose estimation model to extract movement markers from joints of the human body^21^. Note that the process of assessing a person’s movement from video data using a pose estimation model is referred to as the motion capture process in this work. Alpha Pose was favored over other frameworks, such as Open Pose^22–25^, due to its ease-of-use and superior performance on benchmark datasets commonly used in pose tracking^21^. The Alpha Pose pipeline works in a two-step process, which comprises at first a person localization step, using YOLOV3^26^ and EfficientDet^27^, and subsequently a pose estimation step, while the latter utilizes ResNet^28^ as its backbone model. After applying the pose estimation model, the standard 17 motion markers were extracted corresponding to 17 body parts (e.g., left ankle, right hip) according to the COCO keypoint format (see Supplementary Table 2 for details). The data provided by Alpha Pose yields the horizontal and vertical positions of each marker in each frame of the video. Further on, from the Alpha Pose output, 4 multivariate time series were extracted, where each frame was considered a time step. (1) The first time series was based on the raw x-positions of the hip, wrist, and ankle (each left and right, respectively), which were assembled into a 6-dimensional (2 + 2 + 2) time series from here on referred to as *X-pos*. (2) Furthermore, distances between pairs of markers were extracted and formed the 6-dimensional time series, here referred to as *Dist*. The elements of *Dist* are the distances between both ankles, between both wrists, and the distances between the left hip and left wrist, between the right hip and right wrist, and the distance between the neck and left hip, and neck and right hip. (3) & (4) Finally, triples of markers from the upper and lower limbs were used to form triangles, and time series were based on the extracted angles. *Upper* consists of the angles at the shoulders, formed by the triangle shoulder, wrist, and hip, left and right, respectively. *Lower* comprised the angles at the hips, formed by the triangle between both ankles and the left and right hip, respectively. Thus, two 2-dimensional time series resulted for *Upper* and *Lower*. In summary, time series of movements were generated such that each time point corresponds to one video frame. Time series of 6 raw x-positions (*X-pos*), 6 distances (*Dist*), and in total 2×2 triangles (*Upper* and *Lower*) are further analyzed. This data generation is further illustrated in Supplementary Figs. 3–6. Written informed consent to publish the identifiable images shown in Supplementary Figs. 3–6 was obtained from the individual depicted.

### Models and training

We employed two types of feature sets and two distinct machine learning (ML) strategies to reconstruct the SARA gait score, resulting in four model architectures. Each architecture included both a classifier and a regressor. In the first architecture, we combined the Python package tsfresh^29^ with XGBoost^30^. tsfresh extracts 794 predefined features from a one-dimensional time series, such as variance, number of local maxima, and other statistical and signal-based metrics. For multivariate time series data used in this study (*X-pos*, *Dist*, *Upper*, *Lower*), we obtained N × 794 features, where *N* corresponds to the number of time series per category: *N* = 6 for *X-pos* and *Dist*, and *N* = 2 for *Upper* and *Lower*. The extracted features were used to train an XGBoost classifier or regressor, depending on the task. The second architecture also used tsfresh features but replaced XGBoost with ridge regression or classification. Thus, the two tsfresh- based models only differed in their final prediction model. The third and fourth architectures used ROCKET^31^, a time series method that applies random convolutional kernels to extract features. ROCKET has demonstrated state-of-the-art performance in classification tasks while being computationally efficient. In our implementation, 10,000 convolutional kernels were applied to the time series, and ridge regression was used as the final prediction model. For the sake of completeness, we combined ROCKET features with XGBoost classifiers and regressors for the fourth architecture. Since ROCKET requires fixed-length input, we cropped all time series to the first 400 frames, which corresponds to approximately 13 seconds at 30 frames per second. This cut-off was chosen based on the shortest video duration in the dataset and was only applied for ROCKET-based architectures. Each model, defined as a specific combination of feature extractor and ML predictor, was trained and evaluated using the time series categories (*X-pos*, *Dist*, *Upper*, *Lower*) both independently and in all possible combinations. For example, combining *X-pos* and *Dist* (both with dimensionality 6) results in a 12-dimensional multivariate time series. In all cases, we report the best-performing time series or combination thereof. All models were trained for both regression and classification tasks. In regression, the models predicted the continuous SARA gait score in the range [0, 4], excluding HC. In classification, the task was divided into 15 binary problems, each distinguishing between a unique pair from [HC, 0, 1, 2, 3, 4], excluding self-pairs. SARA gait scores were treated as discrete classes, with HC treated as a separate class, in the classification setting. Regression performance was assessed using Root Mean Squared Error (RMSE) and *R*^2^-score, and the best performing model was selected by *R*^2^-score. We performed Mann-Whitney U-tests on predicted values across adjacent classes to evaluate whether models could distinguish neighboring SARA scores. Regression experiments were repeated with HC included for completeness. For classification, the macro-averaged *F*_1_-score was used to account for class imbalance. This score is the average of per-class *F*_1_-scores. We used a modified leave-one-out cross-validation. For participants with follow-up visits, all remaining videos of the same participant were excluded from the training set when one of the videos was part of the test set, to avoid subject-specific learning. Ridge models used inner cross-validation to tune the regularization parameter *α*. For XGBoost models, hyperparameters (learning rate, max tree depth, number of estimators) were optimized via 30 trial runs on the inner fold. Scikit-learn^32^ was used for ridge models due to its convenient interface for tuning *α*. Section lists further information about the implementation and hyperparameter tuning. During the training of tsfresh+XGBoost models, SHAP values were computed for each test sample. These were aggregated across folds, and mean SHAP values were used to rank the time series by feature importance. This offered interpretability of model predictions, in line with current explainable ML practices^33^. Since regression outputs are float-valued, we implemented an additional step to derive discrete predictions for comparison with the human-labeled scores. For each regression prediction, a binary classifier was used subsequently to determine the final class. For example, a prediction of 2.42 would trigger a classifier trained to distinguish class 2 from class 3. To reflect the regressor’s confidence, we dynamically adjusted the classifier’s decision threshold based on the distance of the regression output from the class boundary. For details on this, please see Supplementary - Hybrid Ordinal Regression Implementation. This process was applied to all four models, and the resulting class predictions were used to compare model performance with human baselines.

### Longitudinal analysis

Thirty patients were followed longitudinally, not earlier than 6 months after the previous visit, enabling the evaluation of extracted gait features in capturing progressive gait disturbances over time. For each feature derived using the tsfresh package, we computed the Pearson correlation between the feature value and time (measured in days since the baseline visit). SARA gait scores and time series features were normalized relative to each patient’s baseline, and a linear regression was performed to test the significance of the longitudinal change over time. All p-values from the regression were corrected for multiple testing using the Benjamini-Hochberg method^34^. Analyses were conducted on the full cohort (*N* = 30) as well as four sub-cohorts stratified by baseline SARA gait score. For both, the full cohort and each sub-cohort, the top features are reported based on the highest absolute Pearson’s correlation coefficients. To provide a measure of uncertainty for the correlation estimates, we used bootstrapping (1000 resamples) to compute 95% confidence intervals for Pearson’s correlation coefficients. Additionally, we performed a mixed-effects analysis on the subset of patients with at least one follow-up visit. For each feature within the combined set (*X-pos*, *Dist*, *Upper*, *Lower*), we fitted a linear mixed model, modeling the relationship between the feature and SARA gait score over time^35^. The top three features, ranked by corrected p-values, are reported.

### Fairness analysis

Since this work aims to be incorporated into clinical practice in the future, it was necessary to investigate whether the models resulting from this work are fair. Here, we consider the models as fair if no sex or age group has to expect a significantly lesser performance. Hence, the RMSE for males and females, as well as for the age groups 19–39, 40–59, and 60–82, was calculated and reported. In this analysis, we used RMSE instead of *R*^2^-score because the group sizes vary, and *R*^2^-score is sensitive to sample size.

### Statistics and reproducibility

The statistical analysis conducted in this work uses machine learning models that were trained and evaluated in a cross-validation framework. The dataset consisted of 91 patients with ataxia and 28 healthy controls, with each participant contributing up to three walking videos. No technical replicates were produced, and each data point corresponds to one videotaped clinical assessment. Reproducibility is ensured by applying a deterministic leave-one-out cross-validation with fixed random seeds, and all results are reported in terms of either *F*_1_-score (classification), RMSE and *R*^2^ (regression), and correlation metrics (longitudinal analysis). The clinical setting, pre-processing, feature extraction, and model training, including hyperparameter tuning, are described in the methods to allow replication.

## Results

Table 1 summarizes the demographic and characterizing data of the patient and healthy control cohorts. From 246 videotaped visits, 159 videos of participants performing the normal gait task formed the basis for the following analysis. 87 videos had to be excluded due to given reasons related to video quality or a SARA gait score of greater than 4. The on-site ratings, which serve as the ground truth, were distributed as depicted in Supplementary Table 4.

**Table 1:** Demographic and characterizing data of the cohort

	HC	Patients
N	28	91
f/m	14/14	46/45
^N^videos	30	129
Age	47.75 (19.67)	50.21 (13.80)
SARA sum score	2.59 (3.36)	10.16 (5.26)
SARA gait score	0.36 (0.66)	1.89 (1.07)
INAS count	0.76 (0.76)	2.57 (1.62)

Values are given as mean and standard deviation in brackets. INAS count was available for 139, and SARA sum score was available for 149 videos. Age, INAS, SARA, and SARA gait score are averaged across all videos within the respective group.

*N*_videos_ number of available videos, *f* female, *m* male.

**Fig. 1:**
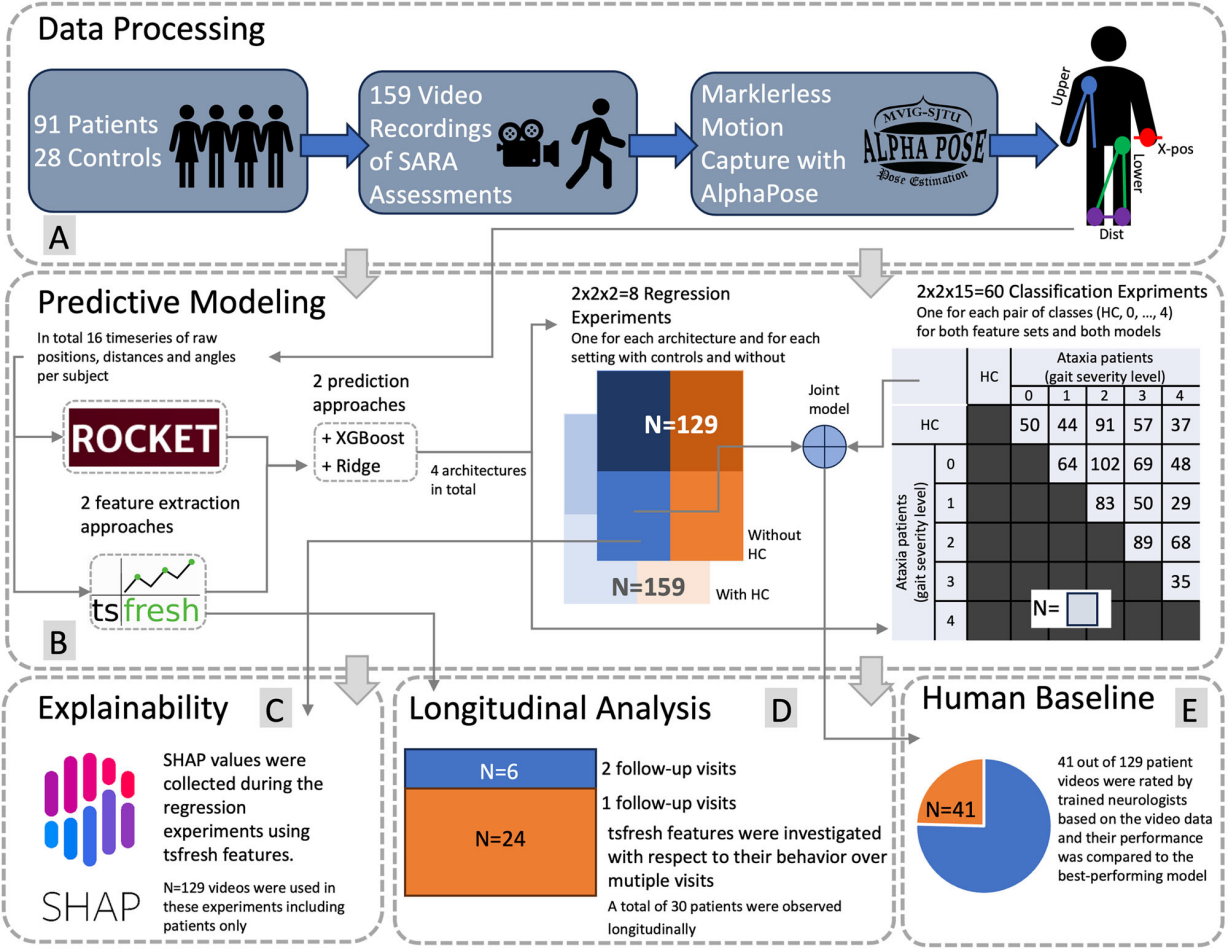
Summary Figure outlining the working steps performed in this study, while indicating the number of samples used in each step. Summary figure depicting the main analyses performed in this study. First, *Data Processing* (**A**) comprises videotaping of normal gait and subsequent motion capture resulting, per subject, in 16 time series of 6 raw x-positions (*X-Pos*), 6 distances (*Dist*), and in total 4 triangles, left and right side of the upper (*Upper*) and lower (*Lower*) limbs. The *Predictive modeling* (**B**) aimed at the reconstruction of the ground truth SARA gait score and compared four different architectures for this purpose. In the *Explainable AI* (**C**) part, we computed SHAP values during the regression experiment using the tsfresh+XGBoost model to identify the most influential time series, which were then interpreted as key parameters for distinguishing between different SARA gait scores. Subsequently, intra-individual trajectories of the tsfresh features were analyzed in the *longitudinal analysis* (**D**) section. We investigated whether time series features are capable of modeling disease progression, potentially more fine-grained than the clinical score. Finally, all models are compared with a *Human Baseline* (**E**) performance. Logos were taken from: [https://github.com/shap/shap, https://github.com/blue-yonder/tsfresh, https://github.com/MVIG-SJTU/AlphaPose, https://link.springer.com/article/10.1007/s10618-020-00701-z]. HC=Healthy Control.

The age distribution across ataxia patient subgroups according to their baseline SARA gait scores and the group of HC is given in Supplementary Fig. 7. We evaluated the performance of the four models described in Section *Models and training* across all time series and their combinations, in both classification and regression settings. Subsequently, this section presents the results of the explainability efforts and concludes with the results of the longitudinal and fairness analysis. For guidance, Fig. 1 illustrates the main parts of the analysis conducted in this work.

### Regression experiments

**Fig. 2:**
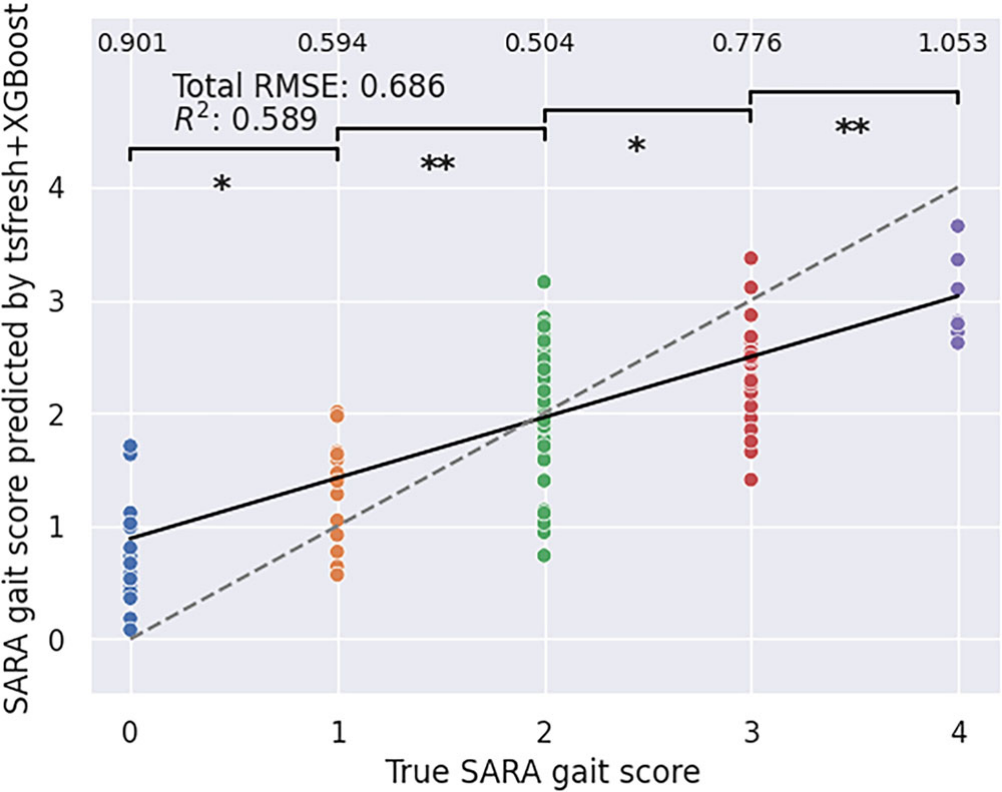
Results of the best-performing regression experiment with the true values plotted against the predicted values alongside the considered performance metrics. Predicted SARA gait values of the best-performing tsfresh+XGBoost regression model were evaluated using root mean squared error (RMSE) and *R*^2^-score. The numbers in the very top row present the RMSE constrained to the respective SARA gait scores. The thick black line is a linear fit on the model predictions, while the dotted line is the diagonal representing a theoretical 1:1 relationship between true and predicted values. The brackets in the top section indicate whether there is a statistically significant difference between the predicted SARA gait scores of neighboring pairs. The significance was tested using a two-sided Mann-Whitney U-Test, and the reported p-values are uncorrected for multiple comparisons. *p < 0.05, ** *p* < 0.01, n.s. = not significant. The exact *p* values are: (0,1): 0.0054, (1,2): 4.138 *∗* 10^*−*5^, (2,3): 0.029, (3,4): 0.0004.

The performance of the four included models in predicting the SARA gait score within patients only (*N*_videos _= 129) was evaluated using RMSE and *R*^2^ metrics, as shown in Fig. 2. The experiments were conducted with every time series combination possible, and the reported scores are the best noted, in terms of *R*^2^-score. Amongst all four models, the tsfresh+XGBoost model, which utilizes explicit time series features, e.g., number of peaks, showed the lowest *RMSE* of 0.686 while reporting the highest *R*^2^-score of 0.589. These scores were reached by utilizing the combination of time series *Dist* + *Upper* + *Lower*, i.e. the body markers taken from the 6 distances between markers combined with the four angles at the left and right hip and shoulder, respectively. Moreover, the model demonstrated a statistically significant (*p* < 0.05) distinction in the distribution of predictions across all pairs of neighboring ratings on the SARA gait scale (Fig. 2). This indicates that the model effectively captured the nuances between adjacent score levels. The remaining three models, tsfresh+Ridge, ROCKET+Ridge, and ROCKET+XGBoost, demonstrated worse performances, and the respective results are provided in Supplementary Figs. 8–10.

All regression experiments were repeated with additionally including HC. Generally, all models perform better in the overall *R*^2^-score when including HC. In this setting, the tsfresh+XGBoost model achieved an *R*^2^-score of 0.638 and an RMSE of 0.705, again making it the best-performing model among the four evaluated. The results of the regression experiments, including control subjects, for all models, are presented in Supplementary Figs. 8-11.

### Classification experiments

#### Binary classification experiments

**Fig. 3:**
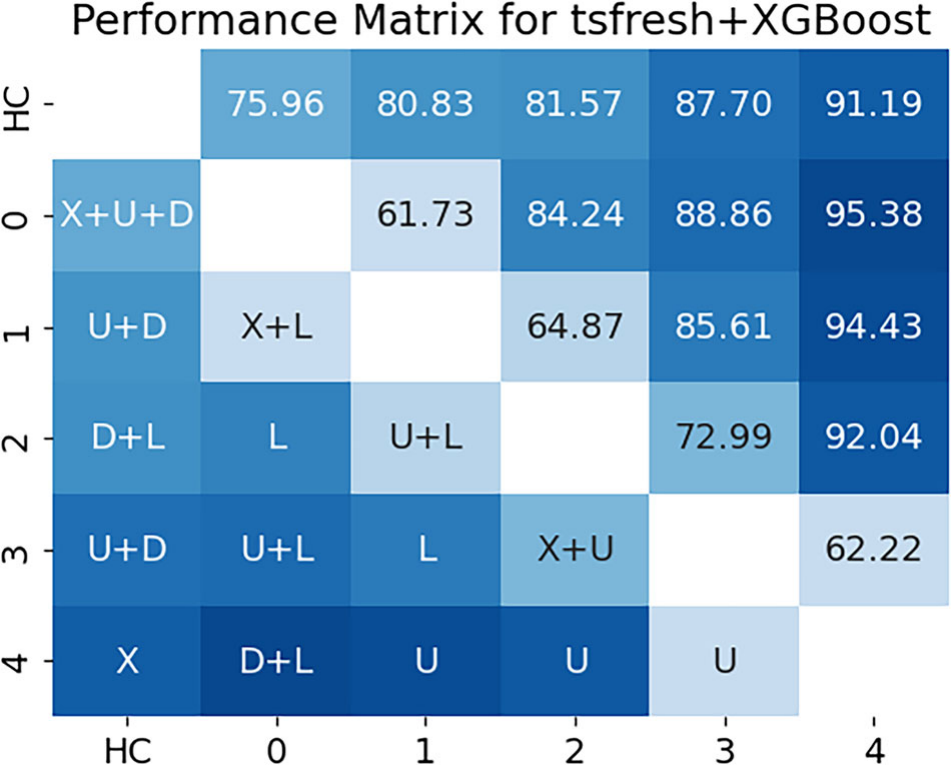
Results of the best-performing classification experiment presented in a color-coded matrix depicting the performance score and the employed time series or combination of such. Results of tsfresh+XGBoost classification model where each row-column combination in the upper right triangle depicts the best reported macro-averaged *F*_1_-score (in %) for the respective binary classification. The lower left triangle depicts for which time series or combination of time series this performance was reported. Classes labeled 0–4 refer to patient data only, while the class labeled HC includes all healthy control subjects. For instance, the best tsfresh+XGBoost model fed with time series features derived from the x-positions (*X-pos*) and angles at the hips (*Lower*) and trained to classify between the SARA gait scores 0 and 1 within the ataxia group was able to score a macro-averaged *F*_1_-score of 61.73%. X = *X-pos* (time series of raw x-positions of each marker separately), D = *Dist* (time series of distances between two markers), U = *Upper* (time series of angles of the upper body part, i.g. shoulders), and L = *Lower* (time series of angles of the lower body part, i.g., hips). HC = Healthy Control.

The classification experiments consisted of 15 binary classification tasks evaluated across four model architectures. Among the models, the tsfresh+XGBoost model ranked among the top performers and was selected for inclusion in the main manuscript. Although it performed on par with the tsfresh+Ridge model in terms of mean macro-averaged *F*_1_-score (81.3% vs. 81.4%, respectively), it had previously exceeded the Ridge model in the regression setting. As such, the tsfresh+XGBoost model was chosen for detailed reporting, while results from the tsfresh+Ridge model are shown in Supplementary Fig. 12. Figure 3 presents the results of the 15 classification experiments using the tsfresh+XGBoost model, visualized as color-encoded matrices. In general, the greater the absolute difference between two SARA gait scores, the more accurately the model distinguished between them. The highest *F*_1_- score (95.38%) was achieved in the classification task separating SARA gait scores 0 and 4, while the lowest (61.73%) was observed when distinguishing between scores 0 and 1 within the patient group. Notably, the model achieved an *F*_1_-score of 75.96% when distinguishing healthy controls from ataxia patients with a SARA gait score of 0. A SARA gait score of 0 indicates an unimpaired gait according to the examiner. The tsfresh+XGBoost model utilizes explicit time series features and consistently performed best when incorporating either the *Lower*, *Upper*, or both time series, corresponding to angles at the hips and shoulders, in 14 out of the 15 binary classification tasks. In addition to the macro-averaged *F*_1_-score presented in the main manuscript, the weighted *F*_1_-score for all 15 binary classification experiments, utilizing the combination tsfresh+XGBoost, is depicted in Supplementary Fig. 13. In contrast, the models utilizing ROCKET features, ROCKET+Ridge and ROCKET+XGBoost, yielded inferior performance, with mean macro-averaged *F*_1_-scores of 76.7% and 74.5%, respectively. Results for these models are presented in Supplementary Figs. 14 and 15. Finally, calibration curves for all 15 experiments using the tsfresh+XGBoost model are depicted in Supplementary Fig. 15.

#### Best-performing model

Among the models evaluated, the tsfresh+XGBoost architecture consistently yields superior performance across all classification and regression experiments. Hence, we focused on this model in the subsequent analyses, and it was selected for hyperparameter reporting. The distribution of hyperparameters across cross-validation folds for this model is shown in Supplementary Figs. 17–20, providing additional insights into its typical configurations.

#### Comparison with human baseline

Finally, this work evaluated the ability of the presented models to classify between all 5 SARA gait classes of patients considered, namely [0, 1, 2, 3, 4], and compared that performance with a human baseline. (Table 2). For 41 participants, this human baseline, the consensus rating of the videos by three trained neurologists^20^, was available. The presented scores are macro-average scores across all classes. The human baseline was able to reconstruct the SARA gait score with a macro-averaged *F*_1_-score of 60.57%, macro-averaged precision of 73.03%, and macro-averaged recall of 66.68%. In comparison, tsfresh+XGBoost achieved better performances in all metrics, in particular a higher macro-averaged *F*_1_-score of 63.99% on the reconstruction of SARA gait scores within the 41 considered videos. This performance was achieved using features extracted from the *Upper* time series, specifically the angles at the shoulders. Full confusion matrices for both tsfresh+XGBoost and the human baseline are presented in Supplementary Fig. 21, alongside additional performance metrics (weighted-F1 and Cohen’s *κ*) in Supplementary Table 5. The models utilizing ROCKET features and the one combining tsfresh features with a ridge regressor and classifier did not perform better than the human baseline. They are presented in Supplementary Table 6.

**Table 2:** End-to-end performance of the best-performing model predicting the SARA gait score on the ordinal scale [0, 1, 2, 3, 4] in comparison with the human performance

Model	Prec. ↑	Rec. ↑	*F*_1_ ↑
tsfresh+XGBoost_*H*41_	74.87	67.17	63.99
Human Baseline	73.03	66.68	60.57

Results of the classification experiment across all patient SARA gait score classes [0,1,2,3,4] evaluated on the subset of the 41 cases, for which the human baseline of consensus video ratings of 3 experts were available. Only the tsfresh+XGBoost model surpassed the human baseline. All scores are presented in %. Arrows indicate the favorable outcome; for all considered metrics, higher values are favorable.

*Prec.* precision, *Rec.* recall, *F*_1_ *F*_1_-score.

### Explainability

**Fig. 4:**
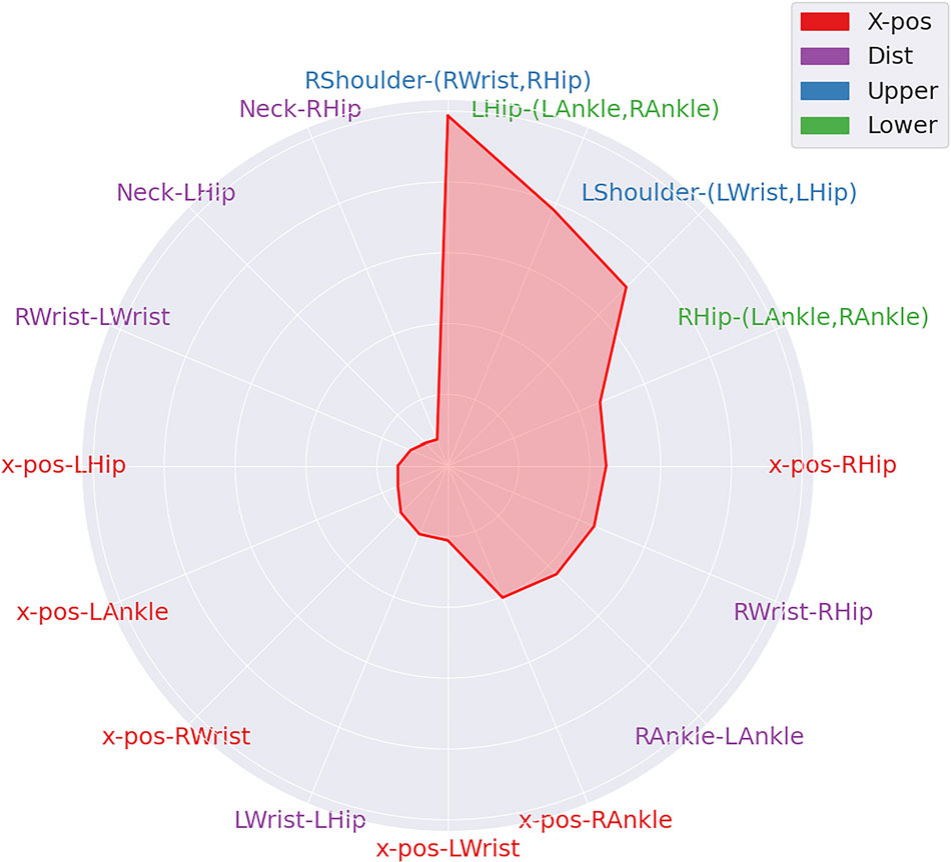
Results of the explainability analysis illustrated in a radarplot with clockwise decreasing importance values beginning at 12 o’clock. Mean SHAP values presented in a radar plot. SHAP values for each feature of the time series of x-pos markers (*X-Pos*), distances (*Dist*), and angles (*Upper*, *Lower*) are calculated during the prediction step of the tsfresh+XGBoost regression model. The respective markers, from which the features with the highest SHAP values originated, i.e., being most important for the final prediction of the model, are arranged in ascending order in a clockwise direction, starting at the 12 o’clock position. R = right, L = left.

Since the implementation of tsfresh+XGBoost involved a mechanism for collecting SHAP values throughout the evaluation process, we utilized these values to gain insight into feature importance. Accumulating these SHAP values allowed us to evaluate which time series were most important for this model to generate its final prediction. Figure 4 presents the results of this investigation in a radar plot. The most important time series comprised the angles of the upper and lower body. The top four time series originated in ascending order from the angles at the right shoulder, left hip, left shoulder, and right hip. The most important time series from the category of time series of raw x-positions (*X-pos*) was derived from the right hip, while the most important time series for distances (*Dist*) was the distance between the right hip and wrist. However, features derived from the raw x-positions of certain body markers (*X-pos*) and from distances (*Dist*) were generally assigned less importance. The same analysis was performed for neighboring classes in the classification experiments using tsfresh+XGBoost. Neighboring classes here refer to the binary classifications HC vs. 0, 0 vs. 1, 1 vs. 2, 2 vs. 3, and 3 vs. 4. Considering the mean SHAP values across neighboring classes, higher SHAP values were most associated with features of the shoulder angles. Detailed results for this analysis are given in the Supplementary Fig. 22.

### Longitudinal analysis

**Fig. 5:**
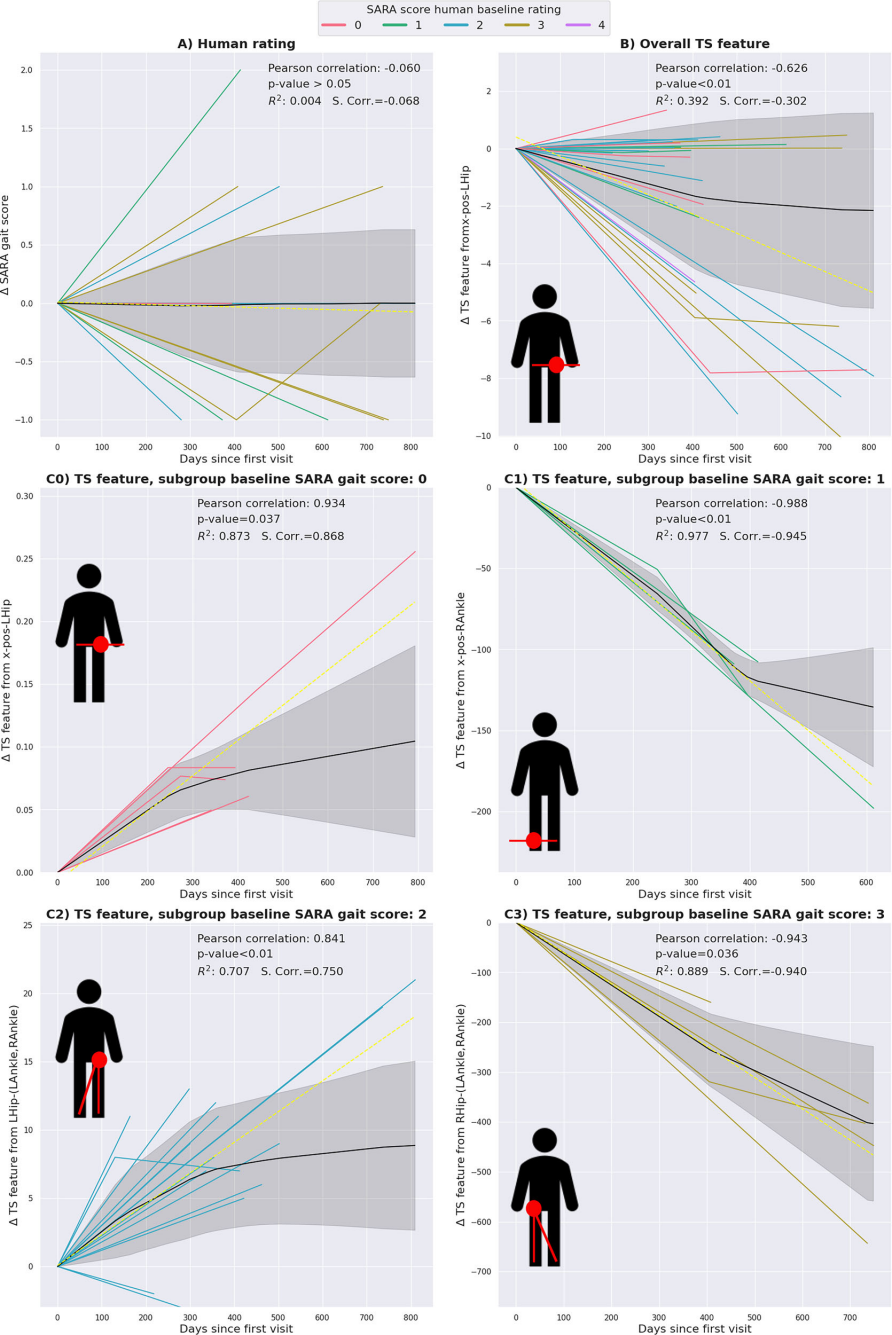
Results of the longitudinal analysis presented in 6 subplots illustrating different markers over time, covering the clinically assessed rating as well as time series markers measured digitally. The relative change to baseline of (**A**) the on-site SARA gait score (human baseline, ground truth, (**B**) the overall, and (**C0**–**C3**) the stage-dependent best features are plotted against time since baseline in days. The anatomical miniature (facing the reader) in the bottom left corner of each time series plot (**B**, C0-C3) indicates from which time series the respective best feature originated. For the stage-dependent analysis, the ataxia patient cohort was divided into subgroups of ataxic gait severity levels according to the baseline SARA gait score of 0 (**C0**), 1 (**C1**), 2 (**C2**), and 3 (**C3**). The black line represents the mean trajectory, created by a linear interpolation, with the gray shaded area being the standard deviation. The yellow dotted line illustrates the linear regression fit of the given data. Trajectories are color-coded according to the baseline SARA gait score. Pearson’s correlation coefficient and a *p*-value, using a Wald test, from the linear regression analysis are given. The p-values are corrected for multiple comparisons using Benjamini-Hochberg. Spearman’s rank correlation coefficient (S.Corr.) and *R*^2^ are shown to provide complementary views on monotonic trends and explained variance. TS = time series, R = right, L = left. The exact p-values for those TS features showing a significant trend are: **B**: 0.00014, **C0**: 0.0374, **C1**: 0.00357, **C2**: 1.346 *∗* 10^*−*5^, **C3**: 0.036.

Thirty participants returned for at least one follow-up visit. Table 3 characterizes this longitudinal sub-cohort. To investigate features suitable for modeling gait disturbances in ataxia over time, we conducted a correlation analysis and visualized the most time-associated parameters, in terms of Pearson correlation, by plotting their relative change from baseline against the number of days since the baseline visit (Fig. 5). Note that the direction of change depends on the specific feature. For example, in one patient tracked longitudinally, one feature extracted from the x-position of the left hip (from the *X-pos* series) decreased over time, while another feature derived from the right shoulder angle (from the *Upper* series) increased. Thus, a longitudinal deterioration of gait disturbances might result in either a decrease or an increase, e.g., negative or positive deltas compared to baseline. A full list of the selected features of the longitudinal analysis is given in Supplementary Table 7.

**Table 3:** Characterizing data of the longitudinal sub-cohort in terms of visits and the respective SARA gait score assessed at the baseline visit

Time of observation in days mean [min, max]	463.43 [130,810]
Number of visits	2 visits: 24
Number of patients according to their baseline level of gait disturbances	3 visits: 6
	SARA gait score of 0: 5
	SARA gait score of 1: 4
	SARA gait score of 2: 15
	SARA gait score of 3: 5
	SARA gait score of 4: 1

The baseline rating refers to the ground truth SARA gait score rating assessed by a neurologist at the first visit. 24 patients had 1 follow-up visit, 6 patients had 2 follow-up visits.

#### Clinical scale

The clinical scale itself, the human rating of SARA gait score, did not show any significant longitudinal changes in the studied cohort. Increases and decreases relative to the SARA gait score at baseline were roughly balanced, whereby the expected deterioration in gait over time was not captured, with a Pearson’s correlation coefficient of −0.06, 95% CI: [−0.431, 0.207], (*p* > 0.05). (Fig. 5A).

#### Overall time series feature

For the overall analysis of the entire ataxia cohort, the feature showing the greatest absolute Pearson correlation with time was derived from the x-position time series of the left hip (Pearson’s correlation coefficient −0.625, 95% CI: [*−*0.778, − 0.421], *p* < 0.01) (Fig. 5B).

#### Stage-dependent time series features

Further stratification of the longitudinal cohort by their SARA gait score rating at baseline allowed for identification of features tailored to the stage-dependent severity of ataxic gait. In each stage-dependent case, the time series feature with the highest Pearson correlation coefficient was statistically significant and showed a stronger association with disease stage than the overall time series feature. (Section) with absolute values of Pearson’s coefficients ranging between 0.841 to 0.988 compared to 0.625 in the overall best feature. For patients that started with a baseline SARA gait score rating of 0, a time series feature of the x-position of the left hip was identified as the best feature for modeling longitudinal changes (Pearson’s coefficient of 0.934, 95% CI: [0.822, 0.986], *p* < 0.01)(Fig. 5C0). For patients with a baseline SARA gait score rating of 1, a time series feature of the x-position of the right ankle best modeled the longitudinal change (Pearson’s coefficient of −0.988, 95% CI: [*−*1.0, − 0.974], *p* < 0.01)(Fig. 5C1). For patients initially rated with a SARA gait score of 2, a feature of the time series from the angle at the left hip (*Lower*) best captured the longitudinal change (Pearson’s coefficient of 0.841, 95% CI: [0.646, 0.944], *p* < 0.01)(Fig. 5C2). For patients who were rated with a SARA gait score of 3 at baseline, a feature from the time series of the angle at the right hip (*Lower*) best captured the longitudinal change (Pearson’s coefficient of −0.943, 95% CI: [*−*0.944, − 0.889], *p* < 0.01)(Fig. 5C3). Since only 1 patient had a baseline SARA gait score of 4, the number of available longitudinal assessments for this severity level was considered insufficient for this analysis. Spearman’s rank correlation coefficient and *R*^2^ are depicted in Fig. 5 for reference without confidence intervals. The linear mixed model analysis, modeling the relationship between time series features and SARA gait score, did not show significant effects (Supplementary Table 8).

### Fairness

Evaluating the RMSE produced by the best-performing regression model, tsfresh+XGBoost, on different age and sex groups gives insight into the fairness of the model. The model performs best applied to the age group 60 to 82 (RMSE: 0.694) and worst for participants aged 19 to 39 (RMSE: 0.744). The age group 40 to 59 ranked between the two other groups (RMSE: 0.714). Concerning the split male/female, the model produced an RMSE of 0.634 for males and 0.751 for females. The results are presented in Table 4. In summary, the tsfresh+XGBoost regression model did not show relevant performance increases or decreases in any sex and/or age group.

**Table 4:** Results of the fairness analysis depicting performance results for different demographic sub- groups

	tsfresh+XGBoost RMSE
Age-range/*N*	
19–39/35	0.744
40–59/59	0.714
60–82/34	0.694
Sex/*N*	
male/59	0.634
female/60	0.751

Root mean squared error (RMSE) of the best-performing regression model, namely tsfresh+XGBoost, for the age and sex groups characterized as given in the left column.

## Discussion

We analyzed videotaped clinical assessments of a normal gait task, including half turn and way back, as part of a clinical assessment in a cohort of ataxia patients and healthy controls. A sensor-free motion capture model, Alpha Pose, was used to create time series by extracting positions of particular body markers, as well as distances and angles between them, in every frame of the video. We applied multiple machine learning (ML) methods and were able not only to reproduce the human clinical scale rating but modestly surpass the human rating in the assessment of subtle and longitudinal changes. In general, the predicted (floating point) SARA gait values of the best-performing model, tsfresh+XGBoost, showed a strong correlation with the human SARA gait rating. Binary classification between two severity levels of gait disturbances in ataxia patients improved on average for more distant classes, i.e. a greater difference between the two considered SARA gait scores. This observation was expected, since it is quite obvious that time series derived from a mildly and a severely ataxic gait vary. However, beyond that, there are two notable aspects with regard to the superiority of the developed automated gait analysis to detect subtle changes that occur in the early course of the disease. First, within the classification experiments, the binary classification between HC and ataxia patients rated by the human examiner as unimpaired, with a SARA gait score of 0 (unipairmed normal gait, unimpaired tandem walk), achieved higher macro-averaged *F*_1_-scores than the binary classification between human SARA gait score rating of 0 (unimpaired normal gait, unimpaired tandem walk) and 1 (unimpaired normal gait, but impaired tandem walk) in ataxia patients. Such participants appeared to have overall time series features of gait that are more similar to those of patients with already impaired tandem walk than compared to those of HC. Moreover, the *R*^2^-score was slightly stronger in the entire cohort comprised of ataxia patients plus HC than for the ataxia patients only. Thus, including HC, with a majority of SARA gait scores of 0, might overall reduce variances within the group of participants rated with a SARA gait score of 0. We hypothesize that both observations are related to the fact that ataxia patients, even if rated as 0 (unimpaired gait in both tasks) by the human examiner, might nonetheless already exhibit very subtle alterations within their gait parameters. From a conceptual point of view, a gradual deterioration is reasonable, and particularly for hereditary ataxias, it has been demonstrated that the manifest stage of the disease is preceded by a biomarker stage with already ongoing and measurable neurodegeneration^36^. Alterations of gait parameters, assessed with body-worn sensors^37^ or a 6-camera multi-Kinect system^38^, have been described for such early, pre-ataxic disease stages. Second, the tsfresh+XGBoost model showed modestly improved performance over the human rater in the discrimination accuracy of SARA gait score 0 (normal gait in both tasks: normal walking and tandem walk)^4^ and SARA gait score 1 (”slight difficulties only visible when walking 10 consecutive steps in tandem”)^4^. In our setup, the second task of the gait assessment, the tandem walk, was not videotaped. Consequently, a human rater by definition would not be able to discriminate SARA gait scores of 0 and 1 based on the normal gait task only. However, the automated analyses achieved a macro-averaged *F*_1_-score of around 60% in this particular binary classification. This again underlines the potential of the presented sensor-free video-based analysis of movement for the detection of subtle, early gait disturbances. Incorporating machine learning models into clinical practice benefits from models being able to reason about their decisions. This not only allows clinicians to gain insight into the importance of certain features leading to the models’ final decision but also increases the trust in these models^39^. The superior performing model, including tsfresh, allowed for the inclusion of an explainability approach in which the time series derived from the considered body markers are ranked by SHAP values. This “explainable AI” analysis revealed that for the overall prediction of SARA gait scores, the top 4 time series with the greatest influence on the model’s final prediction were obtained from the angles at the shoulders and hips. This seems reasonable since the ataxic gait is characterized by a widened base, staggering, and compensation of truncal instability with balancing movements of the arms. A reasoning of the human examiner about the rater’s assigned SARA gait score has not been noted. It is worth discussing whether, in future setups, ‘hand-crafted’ features, which would allow following a rater’s decision, could be included. Such features could be, for instance, of the form: ‘Number of left arm strike outs’ or’asymmetry in the lower limbs’. Ultimately, future experiments could evaluate how well ML models perform in reconstructing the SARA gait score when fed with such additional features, which are currently not assessed within the clinical scale. Beyond being used for predictive modeling, the extracted time series features were also leveraged to track longitudinal progression. To evaluate the sensitivity of the tsfresh time series features to longitudinal change, we assessed how well individual features correlated with time. Among these, the feature showing the strongest association with longitudinal change across all severity levels was the x-position time series of the left hip. It did substantially improve the human rating, which was not able to capture the expected longitudinal deterioration. Notably, analyzing each severity level of gait disturbances separately proved to be even more advantageous. Using features tailored to the actual level of gait impairment at baseline further improved the detectability of progressive longitudinal changes. This finding aligns with previous studies in SCA2, where a 1-year longitudinal change was captured by the digital assessment using three body-worn sensors but not by the clinical scale^9^. While these results are promising, they should be interpreted with caution due to the limited size of the longitudinal cohort.

In addition to the superiority in detecting subtle and longitudinal changes, the automated analysis was more accurate in the reproduction of on-site ratings compared to a human baseline. The reproduction of the on-site rating based on a consensus rating of the videos by three neurologists (referred to as human baseline) did show a slightly lower macro-averaged *F*_1_-score than the best-performing ML model (60.57% vs. 63.99%). We have defined the on-site SARA rating of an experienced human examiner as ground truth. The clinical scale, SARA, has been shown to have a high inter-rater reliability^40,41^. The relatively low *F*_1_-score achieved by the human baseline may be partly attributed to their limited perspective, having access only to the front-facing video recordings. In contrast, the on-site examiner benefits from an optimal viewing position, allowing for a more accurate assessment of participants’ gait. However, the possibility of a subjective bias of the on-site investigator due to the general impression gained during the entire clinical visit cannot be ruled out. Nonetheless, previous studies in smaller cohorts of ataxia patients have demonstrated general accordance of *a posteriori* ratings of videotaped SARA assessments and the respective on-site rating^41,42^. Still, these studies relied on more permissive evaluation metrics, such as the intraclass correlation coefficient (ICC), which are less sensitive to small deviations between predicted and true values. Importantly, ICC is a reliability metric for continuous outcomes and is not designed for evaluating classification performance. In contrast, the macro-averaged *F*_1_-score used in our analysis penalizes all misclassifications equally, without accounting for the ordinal distance between classes. This makes the macro-averaged *F*_1_-score a more class-balanced and stringent metric for assessing predictive accuracy, particularly in settings with class imbalance. The impact of camera positioning on predictive power for both human and ML agents remains speculative here, but might be considered as an object of investigation in future work.

Given the setting presented in this work, we aimed to rule out further potential limitations and biases that may enable the model to make predictions without truly learning the underlying patterns. One of the main risks of such shortcut learning may arise from the age of the participants. However, age and SARA gait score are weakly associated, with an *R*^2^-score below the one achieved by the applied regression model, which allows to conclude that age does not lead to a shortcut learning phenomenon in the given regression scenario (see Supplementary Fig. 23 for *R*^2^-score). Certain groups defined by SARA gait scores showed significant age differences, for instance, between scores of 0 and 1. However, no consistent pattern emerged linking age differences to classification performance (see Supplementary Fig. 24 for *t*-test results). A general limitation of our study is the selection of pose estimation and ML models. We used one pose estimation method, and four time series models were evaluated, with each two utilizing the same input features. Naturally, this narrows the view of the absolute potential of machine learning models in addressing the given task of assessing gait disturbances in ataxia. However, our aim was first of all to investigate whether the rating by a human examiner in a clinical context can be replicated by a given sensor-free, video-based automated movement analysis and whether such a digital assessment may offer further advantages, rather than a methodological comparison of different approaches in a broader sense. Accordingly, the presented work shows in a fundamental first approach the usability and superiority of sensor-free digital biomarkers in the given, limited setting. Future studies will allow for further development of the methodological settings, e.g., by comparing different pose estimation models and/or ML approaches. In addition, future work will explore alternative modeling strategies potentially better exploiting the ordinal nature of the SARA gait score, such as pure ordinal regression, ordered classification, or ranking approaches, which may further improve prediction accuracy and clinical interpretability. Based on this initial work, we see several possibilities for further improvement: incorporating more longitudinal data points, focusing on specific ataxia subtypes, including a larger number of healthy controls across the lifespan, and building a more robust dataset with an optimized ground truth, ideally through on- site assessments involving multiple raters, inter-rater comparisons, and rater consensus. Incorporating more longitudinal samples is essential to validate and strengthen the promising findings regarding the correlation between digital gait features and disease progression. Including multiple raters for the on-site assessment holds the potential for improved ground truth labeling, which ultimately may lead to more accurate models. This might allow to achieve further improvements in the prediction and mapping of biological and pathological movement patterns and provide further insights. Additionally, while a detailed misclassification analysis was beyond the scope of this work, we acknowledge that errors between adjacent SARA gait scores may hold clinical importance in both directions, under- and over-scoring. Future studies should explore these patterns to better understand their potential implications for patient monitoring and care.

We have chosen a very simple test setup with a single camera positioned in front of the participant’s walking distance, which is suitable for fast and easy recording during clinical routine as well as for home recordings. Studies with single-camera recordings at home with a shorter walking distance have already demonstrated a good correlation with the established distance of 10 m investigated here^43^. Interestingly, the study by *Grobe-Einsler, M.* et al. was also able to document the daily form-dependent fluctuations often reported by ataxia patients. The digital methods presented in our work allow large amounts of data to be analyzed in a short time, making it possible to evaluate large numbers of recordings. Therefore, they have enormous potential for overcoming the dependence on individual assessments in the clinical setting, which may be influenced by the current form of the day, as well as for frequent therapy monitoring at home. However, even in the relatively uniform on-site setting of our study, we had to exclude a subset of videos due to disturbed motion capture processes, e.g., due to mirroring surfaces at the walls. This needs to be particularly taken into account for home recordings, which are a strong motivation for digital assessments but remain challenging due to high variability in background and available walking distance. Hence, any efforts utilizing the presented approach have to ensure a certain standard of videotaping and subsequent quality control that allows the motion capture model to assess the subjects’ movements properly. Finally, some methodological considerations are worthwhile to be taken into account within the context of home recordings. As mentioned above, the overall best-performing architecture in this study yielded the combination of tsfresh and XGBoost, incorporating also the opportunity to gain insights into the most important features in an explainable AI approach. However, one big advantage of the alternative ROCKET-based architecture is its low computational costs. This would even make it possible to run ROCKET locally on a smartphone device, providing an elegant way to analyze gait parameters without the need to transfer video data, which can be favorable in terms of data protection.

## Conclusion

Applying machine learning to clinically assessed video data of a simple gait task using sensor-free motion capture and fitting time series models on the extracted markers, as demonstrated in this study, is a promising approach for the automated rating of ataxic gait disturbances. In particular, subtle early and longitudinal changes, not observable by a human examiner, were detected. This method provides an easy-to-use tool feasible for clinical routine as well as for assessments at home, enabling tailored monitoring of disease progression. Further investigation, including the collection of more data, especially longitudinal data from homogeneous disease groups, will help to build even more accurate models. Comparative studies with wearable sensors and the inclusion of additional recording angles are also recommended to enhance the approach.

## Supplementary information

Supplemental material

Supplementary Data 1

## Data Availability

Data is made available upon reasonable request. The data is available in the form of videotaped assessments alongside a table providing the clinical ratings, as well as other characterizing information such as age. All requests shall be addressed to the corresponding author, Philipp Wegner (philipp.wegner@dzne.de). A source data file containing all numerical results underlying the graphs and charts presented in the main figures is available as Supplementary Data 1.

## Code availability

This study did not use any custom code beyond scripts that run the models mentioned in the Methods. The software and the respective versions utilized were the following. The time series features were generated with the tsfresh (v. 0.20.0) framework implemented in Python https://tsfresh.readthedocs.io/en/latest/. The XGBoost models were taken from the Python implementations of XGBoost (v. 1.7.5) https://xgboost.readthedocs.io/en/stable/. The ridge regressor and classifier models were taken from scikit-learn (v. 1.2.2) https://scikit-learn.org/stable/api/ sklearn.linear_model.html. Hyperparameters for the XGBoost-based models were tuned using Optuna (v. 3.3.0) https://optuna.org/. SHAP values were calculated using the SHAP (v. 0.42.1) Python implementation https://SHAP.readthedocs.io/en/latest/index.html. We used the ROCKET implementation provided as a part of sktime (0.36.0) https://github.com/sktime/sktime. Statistical testing was performed using Scipy (v. 1.13.1) https://scipy.org/ Supplementary Table 3 lists the hyperparameter search spaces. Because all models were evaluated in a leave-one-out setting, no single final set of hyperparameters can be reported. To provide insight into typical configurations, we present the distribution of hyperparameters selected during cross-validation for one representative model (tsfresh+XGBoost) in the Supplementary (see Results section for the exact reference).
