# Supplementary material for: Leveraging machine learning for digital gait analysis in ataxia using sensor-free motion capture
Source: Commun Med (Lond). 2026 Jan 27;6:167. doi: 10.1038/s43856-025-01258-y (PMC13031521; doi:10.1038/s43856-025-01258-y)
Supplement: Supplementary file 1 — Supplemental material [file 43856_2025_1258_MOESM1_ESM.pdf]

Supplementary Material - Leveraging machine learning for digital gait analysis in ataxia using sensor-free motion capture

Hybrid Ordinal Regression Implementation

To handle the ordinal nature of our target variable while leveraging both continuous and categorical modeling capabilities, we applied a Hybrid Ordinal Regression approach. This method combines the outputs of a regression model with binary classifiers trained to distinguish between adjacent classes.

Let $y \in \{0, 1, 2, 3, 4\}$ denote the true ordinal labels. The hybrid approach proceeds in two stages:

1. **Regression Prediction:** A regression model $R(\cdot)$ is trained to predict a continuous value $\hat{y}_{\text{reg}} \in [0, 4]$ from the input features.

2. **Classifier Refinement:** For each test input, a binary classifier $C_k(\cdot)$, trained to distinguish class k from class $k+1$, is used to resolve the final label assignment, where $k = \lfloor \hat{y}_{\text{reg}} \rfloor$.

   Let:

   - $f = \hat{y}_{\text{reg}} - \lfloor \hat{y}_{\text{reg}} \rfloor \in [0, 1]$ be the fractional part (i.e., the proximity to the upper class),
   - $p = C_k(x) \in [0, 1]$ be the classifier's predicted probability for class $k+1$,
   - $w \in [0, 1]$ be a tunable hyperparameter controlling the influence of the regression prediction.

   The classification threshold θ is dynamically adjusted as:

   $$\theta = 0.5 + w \cdot (0.5 - f)$$

   The final predicted class $\hat{y}$ is then:

   $$\hat{y} = \begin{cases} k+1 & \text{if } p > \theta \\ k & \text{otherwise} \end{cases}$$

This formulation ensures that when the regressor predicts a value closer to class k, the classifier needs to be more confident to switch to class $k+1$, and vice versa. The hyperparameter w can be tuned on a validation set, with $w = 0$ corresponding to a fixed threshold of 0.5 (i.e., classifier-only decision), and $w = 1$ allowing full adjustment based on the regressor's confidence. This hybrid strategy enables the model to exploit both the ordinal structure of the target and the nuanced estimates from the regression model, potentially improving classification performance near class boundaries. In this work, we have chosen $w = 0.2$ to weigh the regressor by $\frac{1}{5}$, thereby only marginally adjusting the decision threshold. Further note that the decision threshold refinement was only applied to those models using an XGBoostClassifier head, namely tsfresh+XGB and ROCKET+XGB, since only those models provided class probabilities. For the remaining models, namely ROCKET+Ridge and tsfresh+Ridge, the decision threshold was not adjusted and the models' predictions were directly evaluated, i.e., $w = 0$.

Supplemantary Tables

Supplementary Figures

Study name	ID
DANCER	DRKS00008304
DESCRIBE	DRKS00008301
EFACTS	NCT020695
SPORTAX	NCT02701036
EUROSCA	NCT02440763
Ataxia / HSP	DRKS00035152

Supplementary Table 1: **List of all observational studies including their study ID.**
Several observational studies of hereditary and sporadic ataxias are conducted at the Deutsches Zentrum fü r Neurodegenerative Erkrankungen (DZNE), under the umbrella of the so-called DCAN - DZNE Clinical Ataxia Network. The respective trial numbers are provided here.

**Abbreviation**	**Internal Name**	**Body Part**
0	Nose	Nose
1	LEye	Left Eye
2	REye	Right Eye
3	LEar	Left Ear
4	REar	Right Ear
5	LShoulder	Left Shoulder
6	RShoulder	Right Shoulder
7	LElbow	Left Elbow
8	RElbow	Right Elbow
9	LWrist	Left Wrist
10	RWrist	Right Wrist
11	LHip	Left Hip
12	RHip	Right Hip
13	LKnee	Left Knee
14	RKnee	Right Knee
15	LAnkle	Left Ankle
16	RAnkle	Right Ankle

Supplementary Table 2: **List of all 17 Body markers available and their respective short name.**
17 body parts extracted by Alpha Pose model and their respective short name and abbreviations. The provided short name is used in some figures of the main manuscript.

Hyperparameter	Range	Classifier/Regressor	Evaluation on inner fold
Regularization strength (α)	$[10^{-3}, 10^{3}]$	Ridge	Leave-one-out
Number of estimators/boosting rounds	[50, 500]	XGBoost	5-fold
Learning rate	[0.01, 0.3]	XGBoost	5-fold
Max tree depth (Max depth)	[2, 10]	XGBoost	5-fold

Supplementary Table 3: **Hyperparameter details including search space and for which model and with what evaluation strategy that hyperparameter was tuned.**
Hyperparameters tuned in the inner fold of the leave-one-out cross-validation. The first column shows the actual name of the hyperparameter as they are typically called in the literature. The second column states the exact search spaces given to Optuna to sample values from. The third column names the model that required the respective hyperparameter, while the fourth and final column specifies how one setting of hyperparameters sampled was evaluated, i.e., in a leave-one-out cross-validation or 5-fold cross-validation.

SARA gait score	N
0	41
1	22
2	61
3	28
4	7

Supplementary Table 4: **SARA gait score distribution of the entire cohort.**
Distribution of SARA gait scores throughout the cohort used in this study. The right column depicts the number of participants included in the study who were rated with the respective gait score in the first column.

Model	Features	F1-weighted ↑	Cohen's κ ↑
tsfresh+XGBoost$_{H41}$	U	55.17	43.10
Human Baseline	n.a.	53.18	40.89

Supplementary Table 5: **End-to-end performance of the best-performing model predicting the SARA gait score on the ordinal scale [0, 1, 2, 3, 4] in comparison with the human performance in terms of alternative metrics.** Results of the classification experiment on the SARA gait score classes [0,1,2,3,4], presented in %. $model_{H41}$ indicates that the models were evaluated on the subset of the 42 cases, used to create the human baseline. The 'Features' column indicates which time series were utilized to achieve this score. All scores are presented in %. The F1-weighted score depicts the F1-score obtained by weighting each class by its support. Cohen's κ is a statistical score that measures inter-annotator agreement. Arrows indicate the favorable outcome; for all considered metrics, higher values are favorable. n.a.=not applicable

Model	Features	Prec. ↑	Rec. ↑	F_1 ↑
tsfresh+Ridge$_{H41}$	U	70.37	57.42	59.93
ROCKET+Ridge$_{H41}$	X+U+D+L	49.77	47.53	45.50
ROCKET+XGBoost$_{H41}$	X+D+L	55.75	48.41	50.11
Human Baseline	n.a.	73.03	66.68	60.57

Supplementary Table 6: **End-to-end performance of the non-optimal performing model predicting the SARA gait score on the ordinal scale [0, 1, 2, 3, 4] in comparison with the human performance.** Results of the classification experiment on the SARA gait score classes [0,1,2,3,4], presented in %. $model_{H41}$ indicates that the models were evaluated on the subset of the 42 cases, used to create the human baseline. The 'Features' column indicates which time series were utilized to achieve this score. All scores are presented in %. Prec.=Precision, Rec.=Recall, F_1=F_1-score. Arrows indicate the favorable outcome; for all considered metrics, higher values are favorable. n.a.=not applicable

Cohort considered for longitudinal analysis<br>Corresponding multivariate time series	Best performing feature
Whole longitudinal cohort<br>*X-pos*	x-pos-LHip_ change quantiles_ f agg ”mean”__isabs_True__qh 1.0 _ql 0.4
Subgroup with baseline rating: 0<br>*X-pos*	x-pos-LHip__fourier_entropy__bins_3
Subgroup with baseline rating: 1<br>*X-pos*	x-pos-RAnkle__fft_coefficient_ attr_”angle” _coeff_70
Subgroup with baseline rating: 2<br>*Lower*	LHip-(LAnkle,RAnkle)__number_peaks__n_5
Subgroup with baseline rating: 3<br>*Lower*	RHip-(LAnkle,RAnkle)__fft_coefficient_ attr_”imag” __coeff_8

Supplementary Table 7: **Details of the time series features presented in the longitudinal analysis.**
Time series features referred to in the longitudinal analysis (Section 3.5), which optimally modeled the longitudinal change in terms of the reported correlation metrics. Note that implementations of the features can be found here: https://tsfresh.readthedocs.io/en/latest/text/list_of_features.html

Feature	β	R^2-marginal	R^2-conditional	SE	p-value
x-pos-LHip__linear_trend__attr_”pvalue”	0.254	0.488	0.944	0.056	0.081
RShoulder-(RWrist,RHip)__fft_coefficient__attr_”angle”__coeff_94	-0.240	0.483	0.943	0.056	0.083
LWrist-LHip__fft_coefficient__attr_””angle””__coeff_65	0.228	0.466	0.948	0.053	0.083

Supplementary Table 8: **Results of the linear mixed model analysis as a part of the longitudinal analysis.** SE refers to the standard error. The p-values reported are derived from a two-sided Wald test and were corrected for multiple comparisons using Benjamini-Hochberg. For a definition of the features, we refer to the tsfresh documentation: https://tsfresh.readthedocs.io/en/latest/text/list_of_features.html

1) Gait

Proband is asked (1) to walk at a safe distance parallel to a wall including a half-turn (turn around to face the opposite direction of gait) and (2) to walk in tandem (heels to toes) without support.

0 **Normal, no difficulties in walking, turning and walking tandem (up to one misstep allowed)**
1 **Slight difficulties, only visible when walking 10 consecutive steps in tandem**
2 **Clearly abnormal, tandem walking >10 steps not possible**
3 **Considerable staggering, difficulties in half-turn, but without support**
4 **Marked staggering, intermittent support of the wall required**
5 **Severe staggering, permanent support of one stick or light support by one arm required**
6 **Walking > 10 m only with strong support (two special sticks or stroller or accompanying person)**
7 **Walking < 10 m only with strong support (two special sticks or stroller or accompanying person)**
8 **Unable to walk, even supported**

Supplementary Figure 1: **Definition SARA item gait as it is described in the original SARA publication.** Instruction and rating of the item gait of the Scale for the Assessment and Rating of Ataxia (SARA). Reference: Schmitz-Hubsch et al., *Neurology* **66**, 1717-1720. https://www.neurology.org/lookup/doi/10.1212/01.wnl.0000219042.60538.92 (June 2006).

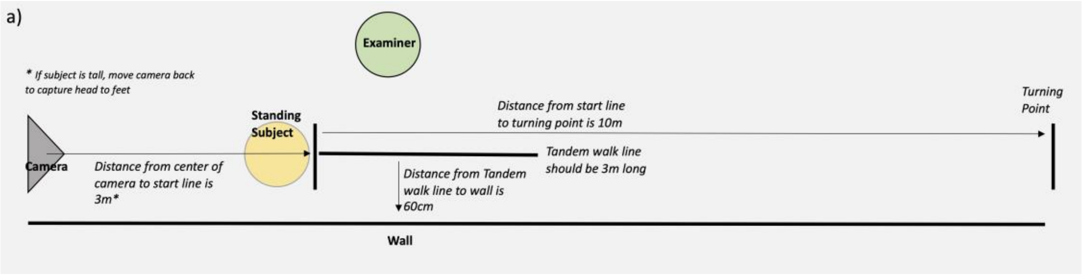

Supplementary Figure 2: **The video protocol used in this study to videotape the SARA gait (sub-)assessment.** Protocol for the SARA video assessments. Figure from: Grobe-Einsler, M. et al. Scale for the Assessment and Rating of Ataxia (SARA): Development of a Training Tool and Certification Program. *The Cerebellum* **23**, 877–880. issn: 1473-4230. https://doi.org/10.1007/s12311-023-01543-3 (June 2024).

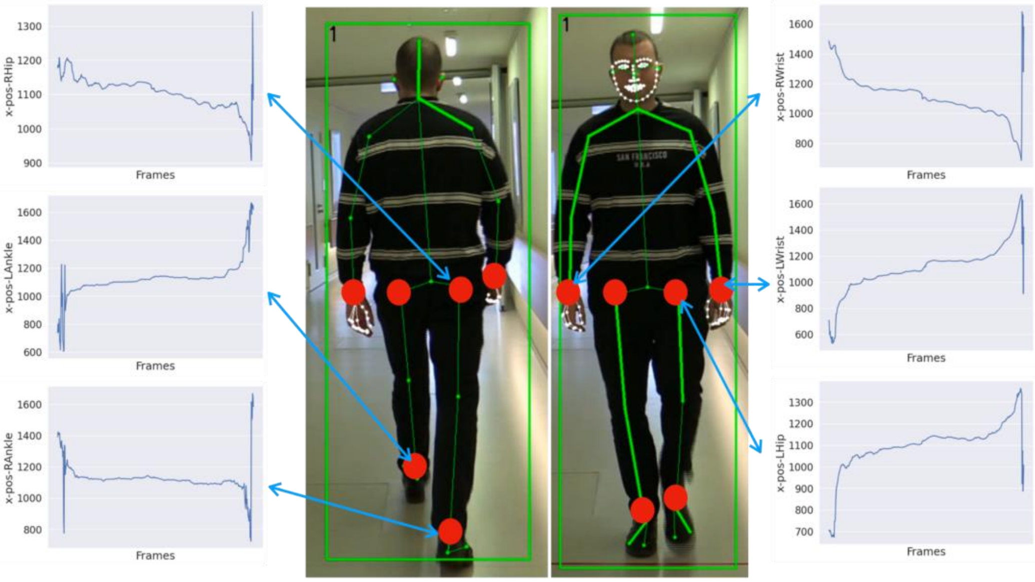

Supplementary Figure 3: **Extraction of *X-pos* markers illustrated for one example subject.**
Illustration of the body markers used to create the time series *X-pos*. The depicted picture originates from the output provided by Alpha Pose. Body markers are annotated by the motion-capturing framework (Alpha Pose). The plots left and right of the image illustrate how the value measured at a certain marker changes over time and further allow to grasp how the time series used in this work looks like.

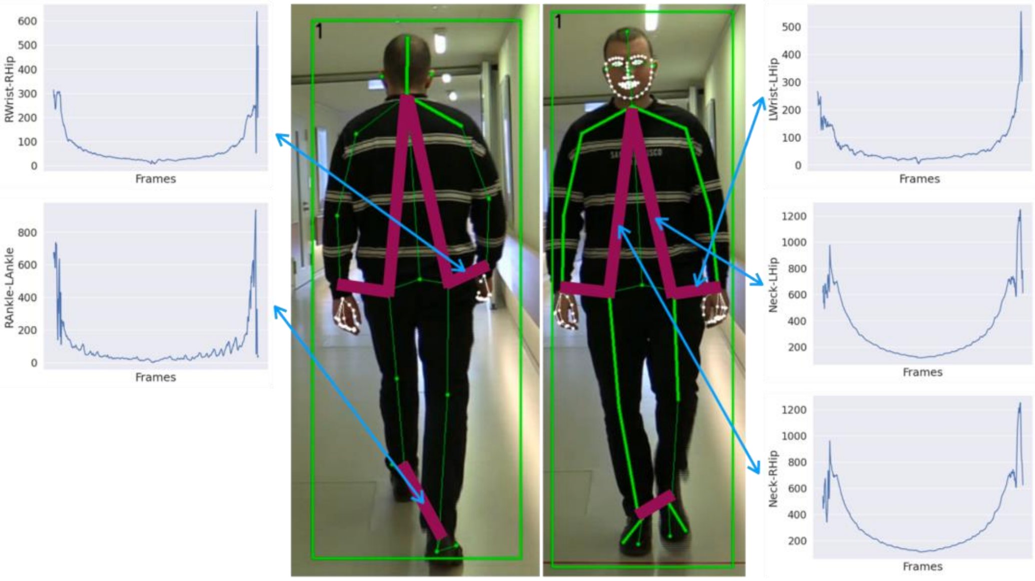

Supplementary Figure 4: **Extraction of the *Dist* markers illustrated for one example subject.**
Illustration of the distances between body markers used to create the time series *Dist*. The depicted picture originates from the output provided by Alpha Pose. Body markers are annotated by the motion-capturing framework (Alpha Pose). The plots left and right of the image illustrate how the value measured at a certain marker changes over time and further allow to grasp how the time series used in this work looks.

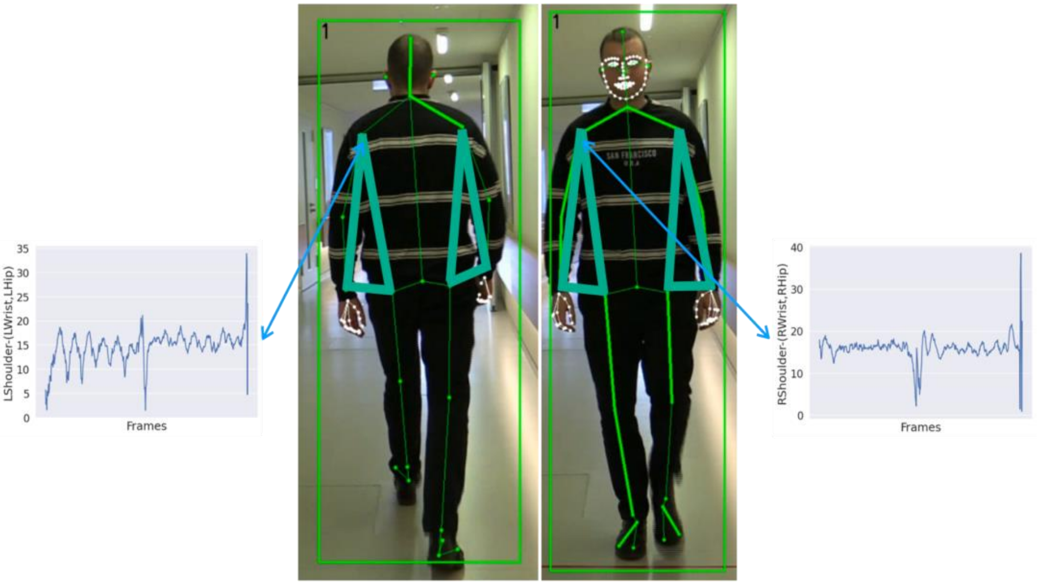

Supplementary Figure 5: **Extraction of the *Upper* markers illustrated for one example subject.**
Illustration of the angles in the upper body used to create the time series *Upper*. The depicted picture originates from the output provided by Alpha Pose. Body markers are annotated by the motion-capturing framework (Alpha Pose). The plots left and right of the image illustrate how the value measured at a certain marker changes over time and further allow to grasp how the time series used in this work looks.

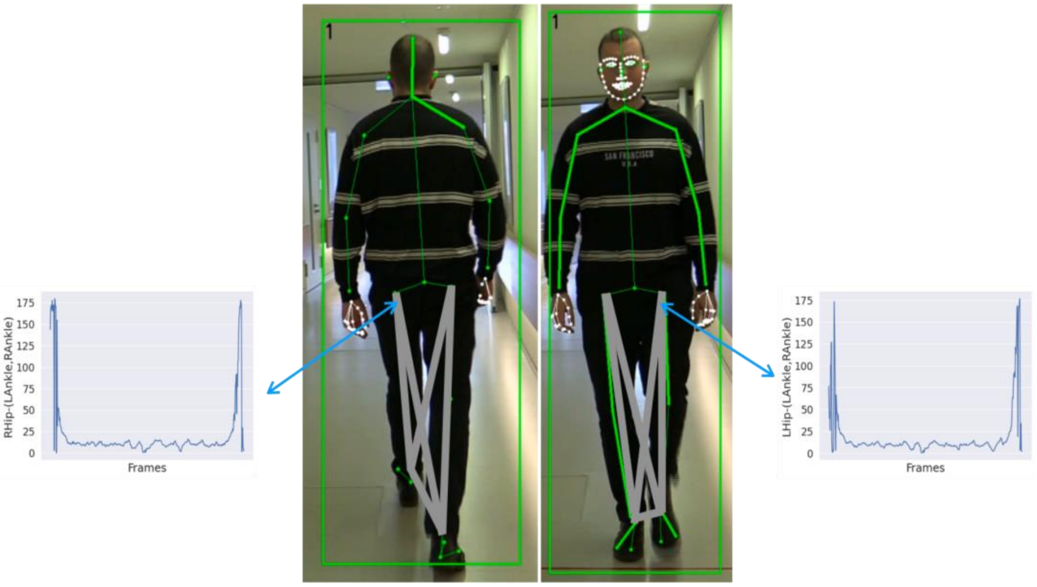

Supplementary Figure 6: **Extraction of the *Lower* markers illustrated for one example subject.**
Illustration of the angles in the lower body used to create the time series *Lower*. The depicted picture originates from the output provided by Alpha Pose. Body markers are annotated by the motion-capturing framework (Alpha Pose). The plots left and right of the image illustrate how the value measured at a certain marker changes over time and further allow to grasp how the time series used in this work looks.

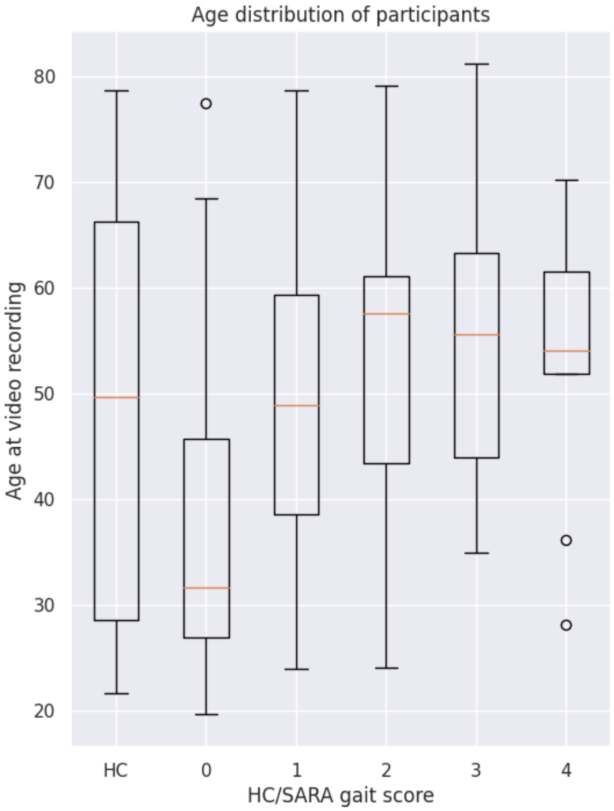

Supplementary Figure 7: **Boxplot of the age across different SARA gait scores and for healthy controls (HC).** Age distributions of participants stratified by the subgroups of HC and for ataxia patients by SARA gait score are displayed in a boxplot.

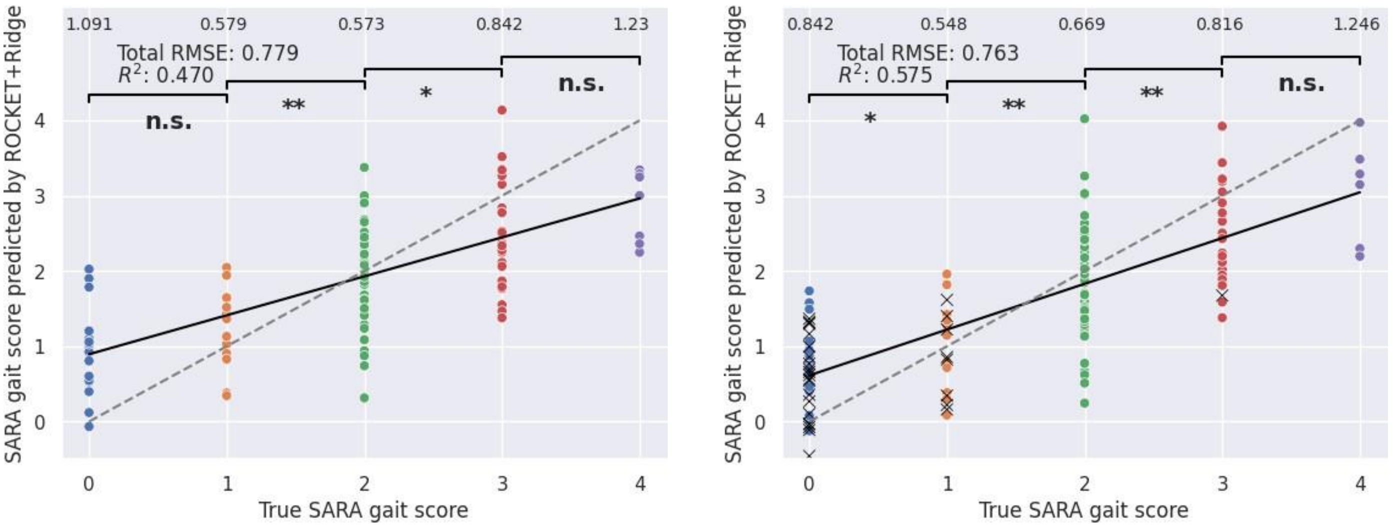

(a) Patients only

(b) Patients+Controls

Supplementary Figure 8: **Results of the ROCKET+Ridge regression experiment with the true values plotted against the predicted values alongside the considered performance metrics.**
Predicted SARA gait values of the ROCKET+Ridge regression model were evaluated using root mean squared error (RMSE) and R^2-score. Both experiments with patients only (a) and with patients and controls (b) are presented. The numbers in the very top row present the RMSE constrained to the respective SARA gait scores. The thick black line is a linear fit on the model predictions, while the dotted line is the diagonal representing a theoretical 1:1 relationship between true and predicted values. The brackets in the top section indicate whether there is a statistically significant difference between the predicted SARA gait scores of neighboring pairs. The significance was tested using a two-sided Mann-Whitney U-Test, and the reported p-values are uncorrected for multiple comparisons. * $p < 0.05$, ** $p < 0.01$, n.s. = not significant. Controls are marked with black crosses. The exact p-values for (a) are: (0,1): 0.28, (1,2): $9.25 * 10^{-5}$, (2,3): 0.001, (3,4): 0.19. The exact p-values for (b) are: (0,1):0.024, (1,2): $8.81 * 10^{-7}$, (2,3): 0.0001, (3,4): 0.087.

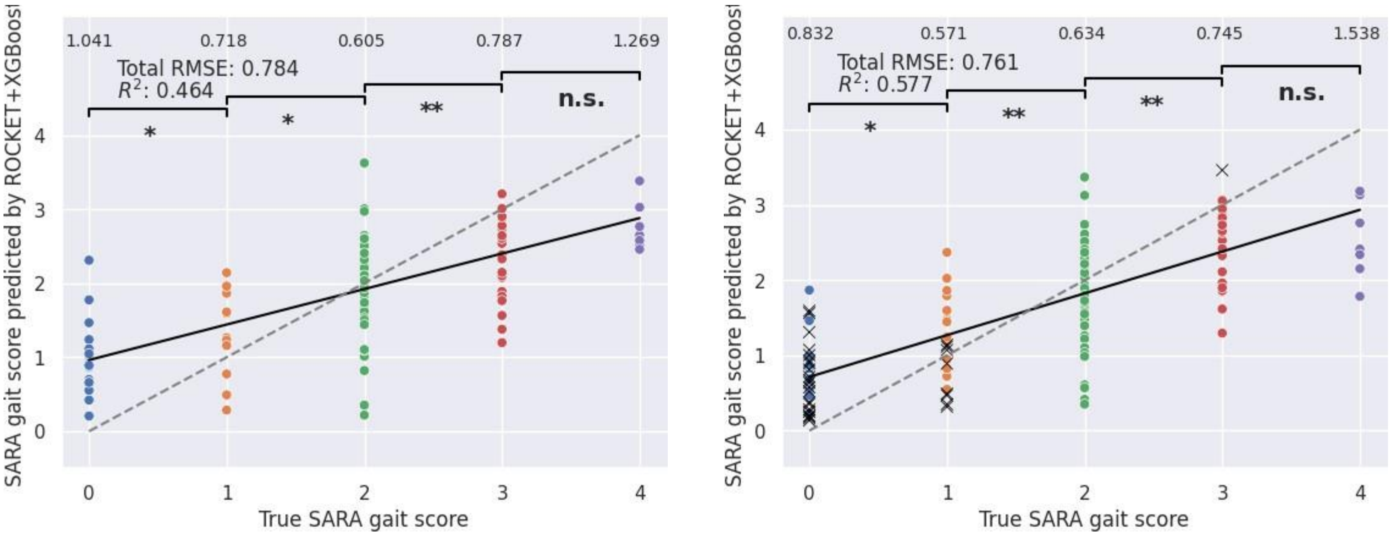

(a) Patients only

(b) Patients+Controls

Supplementary Figure 9: **Results of the ROCKET+XGBoost regression experiment with the true values plotted against the predicted values alongside the considered performance metrics.**
Predicted SARA gait values of the ROCKET+XGBoost regression model were evaluated using root mean squared error (RMSE) and R^2-score. Both experiments with patients only (a) and with patients and controls (b) are presented. The numbers in the very top row present the RMSE constrained to the respective SARA gait scores. The thick black line is a linear fit on the model predictions, while the dotted line is the diagonal representing a theoretical 1:1 relationship between true and predicted values. The brackets in the top section indicate whether there is a statistically significant difference between the predicted SARA gait scores of neighboring pairs. The significance was tested using a two-sided Mann-Whitney U-Test, and the reported p-values are uncorrected for multiple comparisons. * $p < 0.05$, ** $p < 0.01$, n.s. = not significant. Controls are marked with black crosses. The exact p-values for (a) are: (0,1): 0.013, (1,2): 0.01, (2,3): 0.0004, (3,4): 0.17. The exact p-values for (b) are: (0,1):0.006, (1,2): $7.36 * 10^{-6}$, (2,3): $8.27 * 10^{-5}$, (3,4): 0.73.

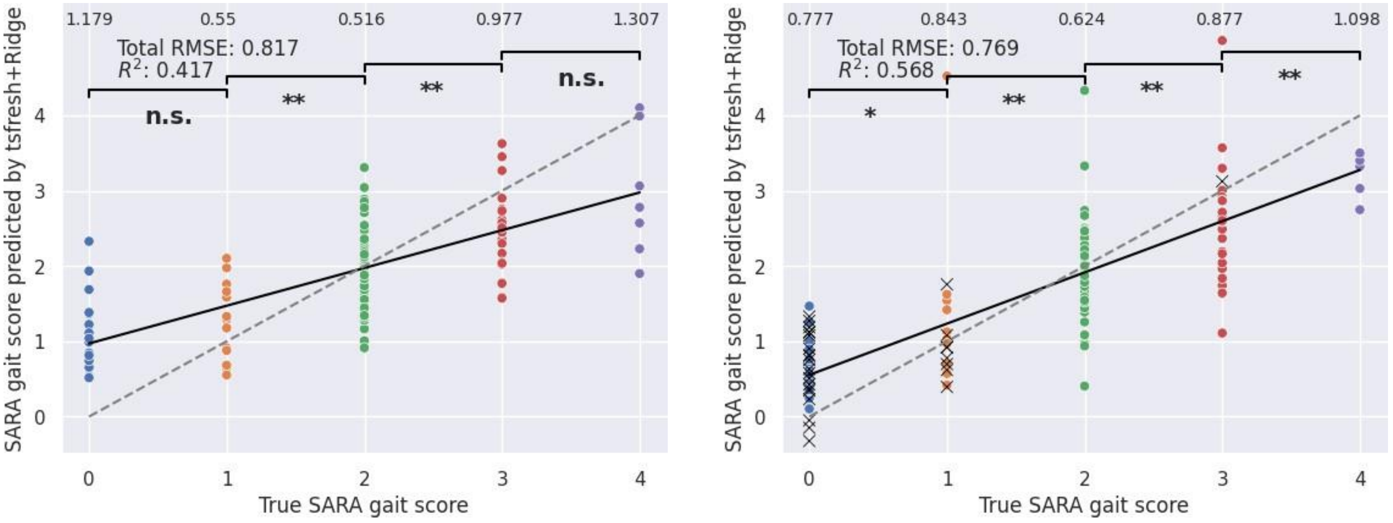

(a) Patients only

(b) Patients+Controls

Supplementary Figure 10: **Results of the tsfresh+Ridge regression experiment with the true values plotted against the predicted values alongside the considered performance metrics.**
Predicted SARA gait values of the tsfresh+Ridge regression model were evaluated using root mean squared error (RMSE) and R^2-score. Both experiments with patients only (a) and with patients and controls (b) are presented. The numbers in the very top row present the RMSE constrained to the respective SARA gait scores. The thick black line is a linear fit on the model predictions, while the dotted line is the diagonal representing a theoretical 1:1 relationship between true and predicted values. The brackets in the top section indicate whether there is a statistically significant difference between the predicted SARA gait scores of neighboring pairs. The significance was tested using a two-sided Mann-Whitney U-Test, and the reported p-values are uncorrected for multiple comparisons. * $p < 0.05$, ** $p < 0.01$, n.s. = not significant. Controls are marked with black crosses. The exact p-values for (a) are: (0,1): 0.28, (1,2): 0.0001, (2,3): 0.0002, (3,4): 0.17. The exact p-values for (b) are: (0,1):0.004, (1,2): $1.27 * 10^{-6}$, (2,3): $2.26 * 10^{-5}$, (3,4): 0.0008.

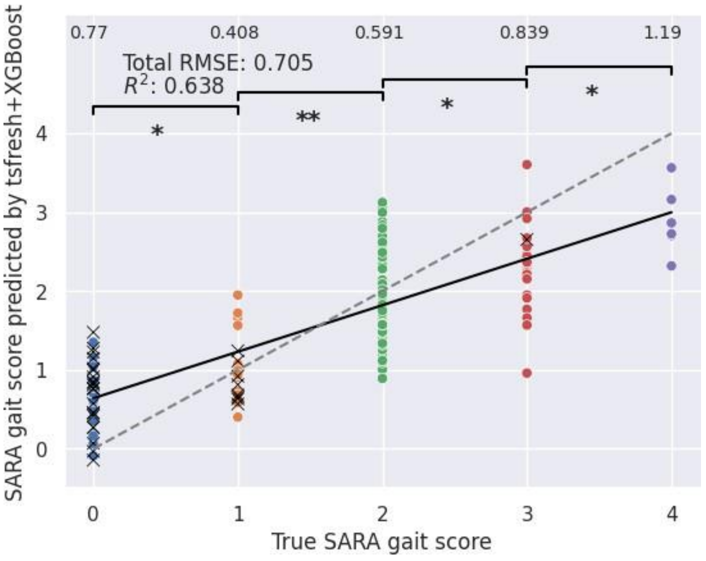

Supplementary Figure 11: **Results of the tsfresh+XGBoost regression experiment trained and evaluated on both patients and controls with the true values plotted against the predicted values alongside the considered performance metrics.**
Predicted SARA gait values of the tsfresh+Ridge regression model were evaluated using root mean squared error (RMSE) and R^2-score. The numbers in the very top row present the RMSE constrained to the respective SARA gait scores. The thick black line is a linear fit on the model predictions, while the dotted line is the diagonal representing a theoretical 1:1 relationship between true and predicted values. The brackets in the top section indicate whether there is a statistically significant difference between the predicted SARA gait scores of neighboring pairs. The significance was tested using a two-sided Mann-Whitney U-Test, and the reported p-values are uncorrected for multiple comparisons. * $p < 0.05$, ** $p < 0.01$, n.s. = not significant. Controls are marked with black crosses. The exact p-values are: (0,1): 0.015, (1,2): $1.12 * 10^{-8}$, (2,3): 0.009, (3,4): 0.003.

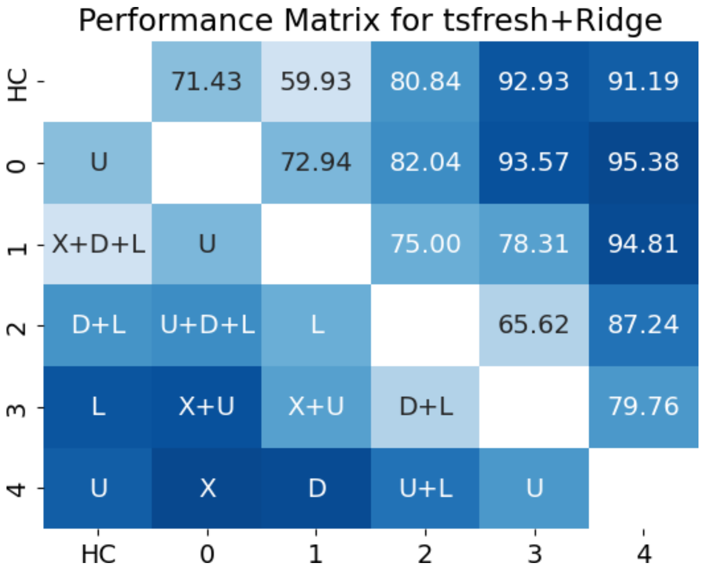

Supplementary Figure 12: **Results of the tsfresh+Ridge classification experiment presented in a color-coded matrix depicting the performance score and the employed time series or combination of such.**
Results of tsfresh+Ridge classification model where each row-column combination in the upper right triangle depicts the best reported macro-averaged F_1-score (in %) for the respective binary classification. The lower left triangle depicts for which time series or combination of time series this performance was reported. Classes labeled 0–4 refer to patient data only, while the class labeled HC includes all healthy control subjects. X=*X-pos* (time series of raw x-positions of each marker separately), D=*Dist* (time series of distances between two markers), U=*Upper* (time series of angles of the upper body part, i.g. shoulders), and L=*Lower* (time series of angles of the lower body part, i.g. hips).

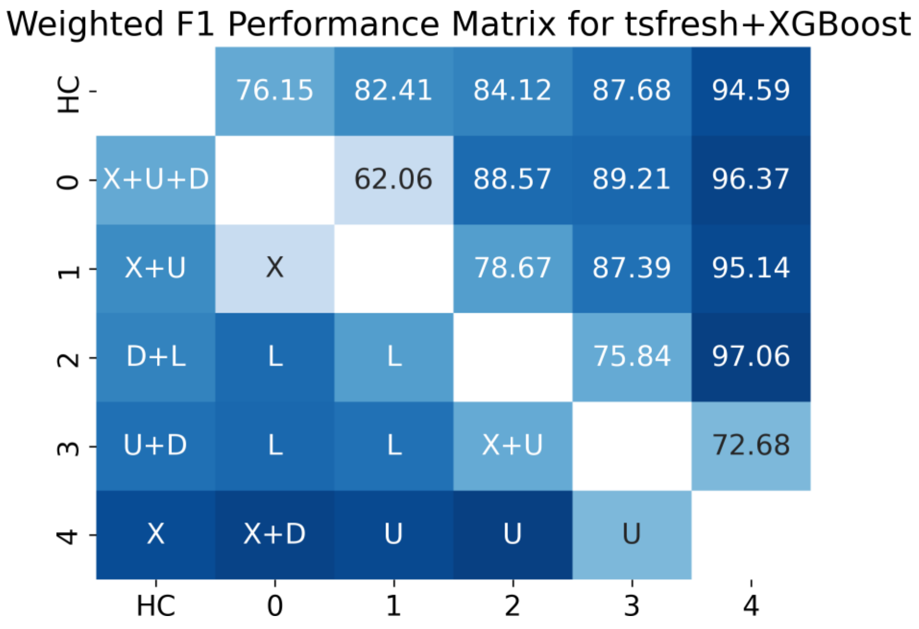

Supplementary Figure 13: **Results of the tsfresh+XGBoost classification experiment, in terms of weighted F_1-score, presented in a color-coded matrix depicting the performance score and the employed time series or combination of such.**
Results of tsfresh+XGBoost classification model where each row-column combination in the upper right triangle depicts the best reported weighted F_1-score (in %) for the respective binary classification. The lower left triangle depicts for which time series or combination of time series this performance was reported. Classes labeled 0–4 refer to patient data only, while the class labeled HC includes all healthy control subjects. X=*X-pos* (time series of raw x-positions of each marker separately), D=*Dist* (time series of distances between two markers), U=*Upper* (time series of angles of the upper body part, i.g. shoulders), and L=*Lower* (time series of angles of the lower body part, i.g. hips). Mean weighted F_1-score: 84.53%.

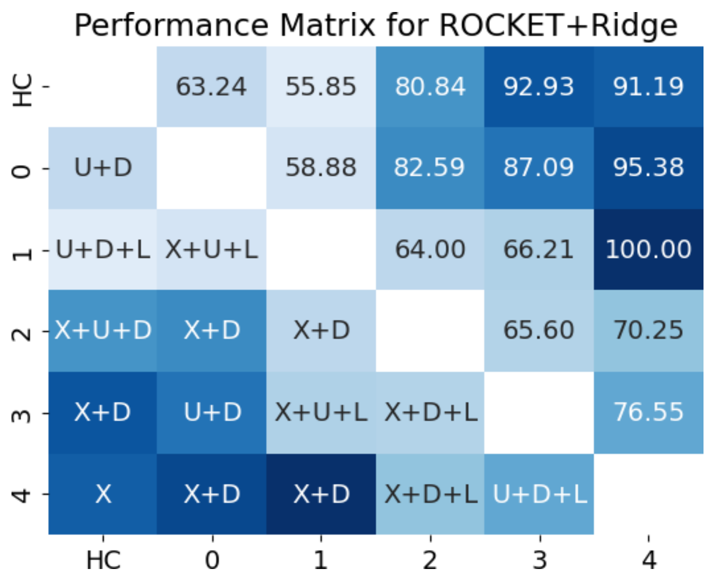

Supplementary Figure 14: **Results of the ROCKET+Ridge classification experiment presented in a color-coded matrix depicting the performance score and the employed time series or combination of such**
Results of ROCKET+Ridge classification model where each row-column combination in the upper right triangle depicts the best reported macro-averaged F_1-score (in %) for the respective binary classification. The lower left triangle depicts for which time series or combination of time series this performance was reported. Classes labeled 0–4 refer to patient data only, while the class labeled HC includes all healthy control subjects. X=*X-pos* (time series of raw x-positions of each marker separately), D=*Dist* (time series of distances between two markers), U=*Upper* (time series of angles of the upper body part, i.g. shoulders), and L=*Lower* (time series of angles of the lower body part, i.g. hips).

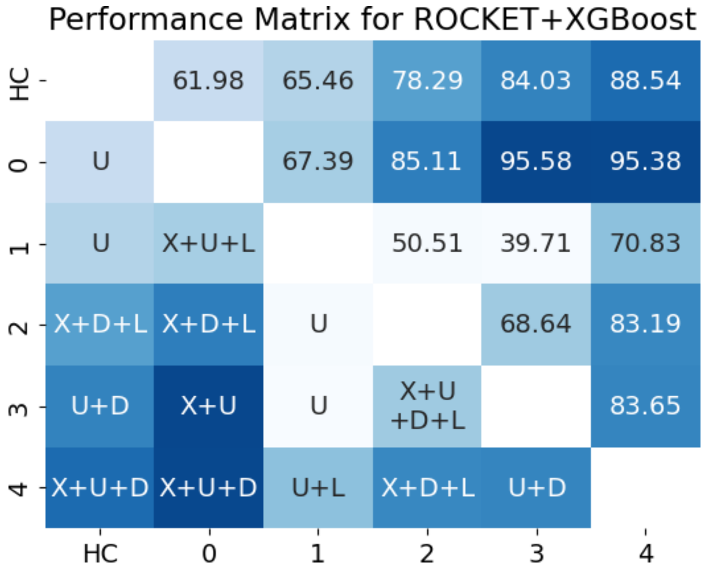

Supplementary Figure 15: **Results of the ROCKET+XGBoost classification experiment presented in a color-coded matrix depicting the performance score and the employed time series or combination of such**
Results of ROCKET+XGBoost classification model where each row-column combination in the upper right triangle depicts the best reported macro-averaged F_1-score (in %) for the respective binary classification. The lower left triangle depicts for which time series or combination of time series this performance was reported. Classes labeled 0–4 refer to patient data only, while the class labeled HC includes all healthy control subjects. X=*X-pos* (time series of raw x-positions of each marker separately), D=*Dist* (time series of distances between two markers), U=*Upper* (time series of angles of the upper body part, i.g. shoulders), and L=*Lower* (time series of angles of the lower body part, i.g. hips).

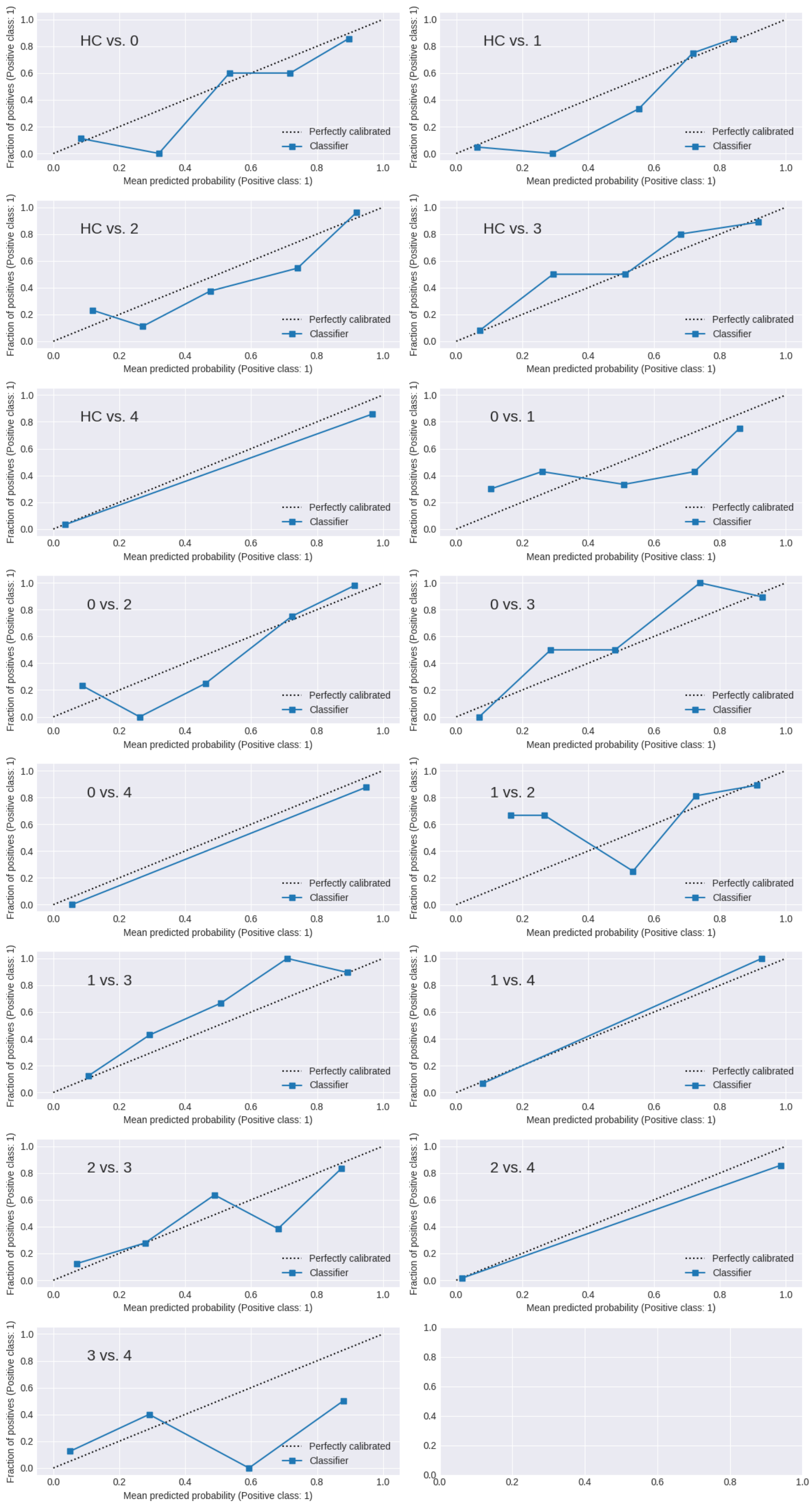

Supplementary Figure 16: **Calibration curves for tsfresh+XGBoost classification experiments.** All calibration curves use 5 bins and have been calculated using *scikit-learn*. For more details, please see: https://scikit-learn.org/stable/auto_examples/calibration/plot_calibration_curve.html

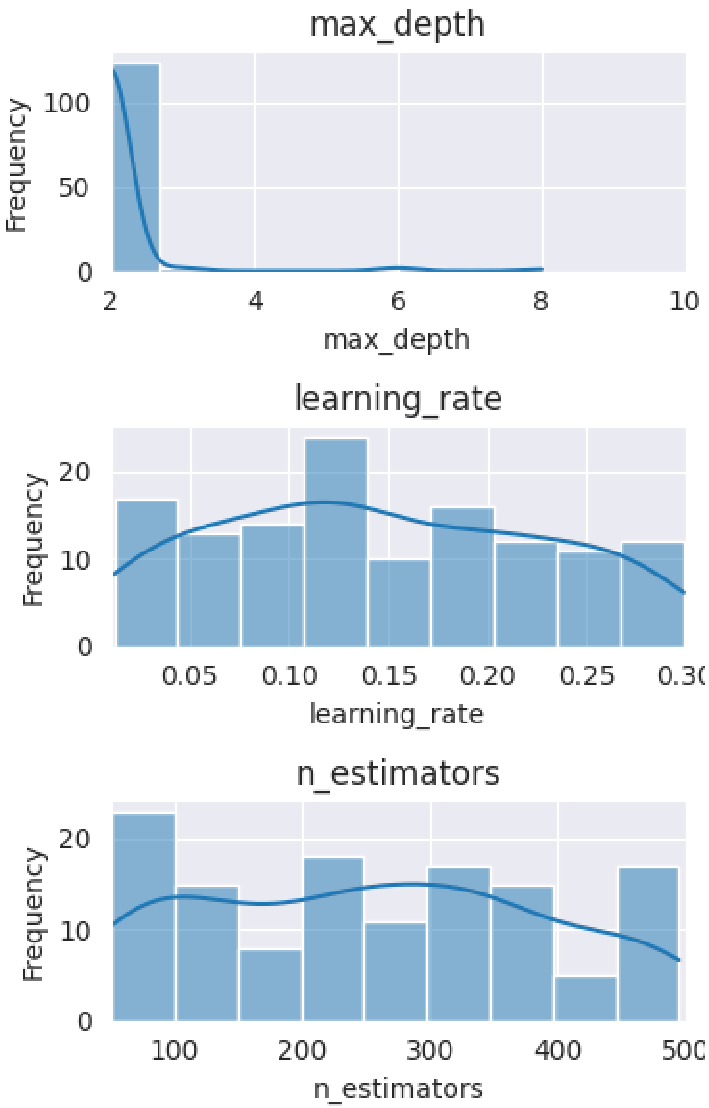

(a) Hyperparameter distribution of the tsfresh+XGBoost regression experiment using patients only.

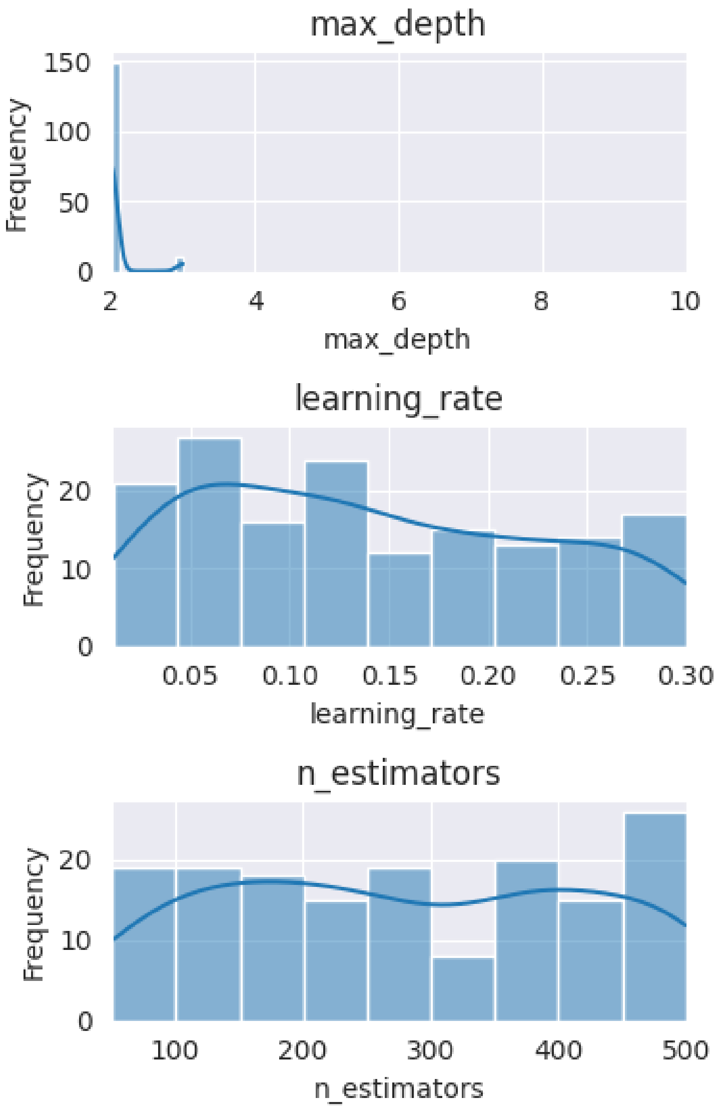

(b) Hyperparameter distribution of the tsfresh+XGBoost regression experiment using patients and controls.

Supplementary Figure 17: **Histogram of hyperparameters during the leave-one-out cross-validation of the tsfresh+XGBoost regression experiment.**
The histograms allow insight into the distribution of favorable hyperparameters during the cross-validation of the best-performing regression model, namely tsfresh+XGBoost. Additionally, each histogram displays a kernel density estimate curve overlaid on top of the histogram bars. The left column (a) of plots depicts the hyperparameter distributions for the experiment considering patients only, while the right column (b) depicts the experiment using patients and controls. Details on the hyperparameter search spaces are given in Supplementary Table 3.

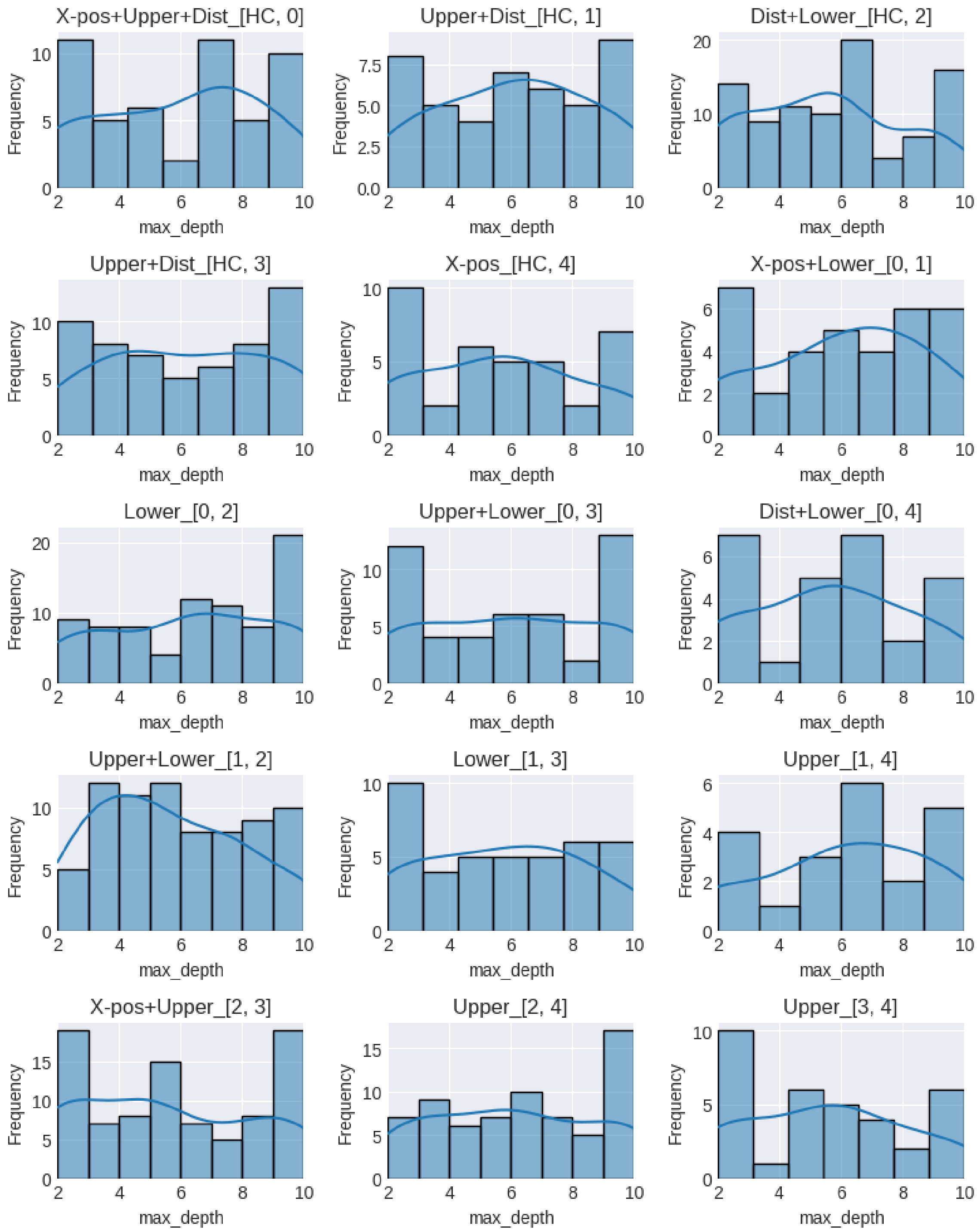

Supplementary Figure 18: **Histograms of max depth hyperparameter during the leave-one-out cross-validation of the tsfresh+XGBoost classification experiment.**
Each plot presents the distribution of the max_depth hyperparameter in a certain binary classification scenario, indicated in the plot title alongside the time series (combination) utilized. For instance, the upper left plot shows the distribution of the hyperparameter for the binary classification trained and evaluated on the classes healthy controls (HC) and SARA gait score 0. The histograms allow insight into the distribution of favorable max_depth values during the cross-validation of the best-performing regression model, namely tsfresh+XGBoost. Additionally, each histogram displays a kernel density estimate curve overlaid on top of the histogram bars. Details on the hyperparameter search spaces are given in Supplementary Table 3.

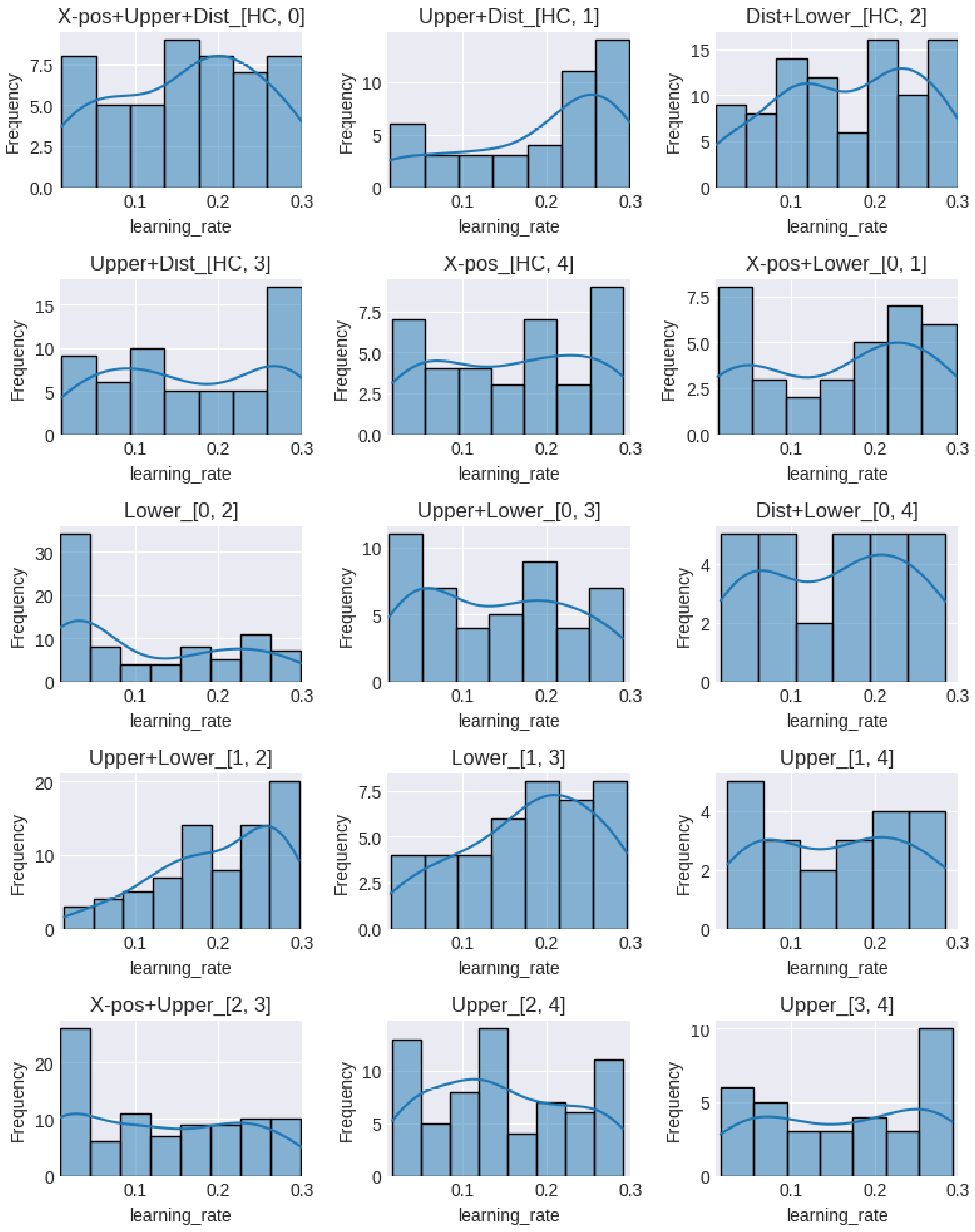

Supplementary Figure 19: **Histograms of learning rate hyperparameter during the leave-one-out cross-validation of the tsfresh+XGBoost classification experiment.**
Each plot presents the distribution of the learning_rate hyperparameter in a certain binary classification scenario, indicated in the plot title alongside the time series (combination) utilized. For instance, the upper left plot shows the distribution of the hyperparameter for the binary classification trained and evaluated on the classes healthy controls (HC) and SARA gait score 0. The histograms allow insight into the distribution of favorable learning_rate values during the cross-validation of the best-performing regression model, namely tsfresh+XGBoost. Additionally, each histogram displays a kernel density estimate curve overlaid on top of the histogram bars. Details on the hyperparameter search spaces are given in Supplementary Table 3.

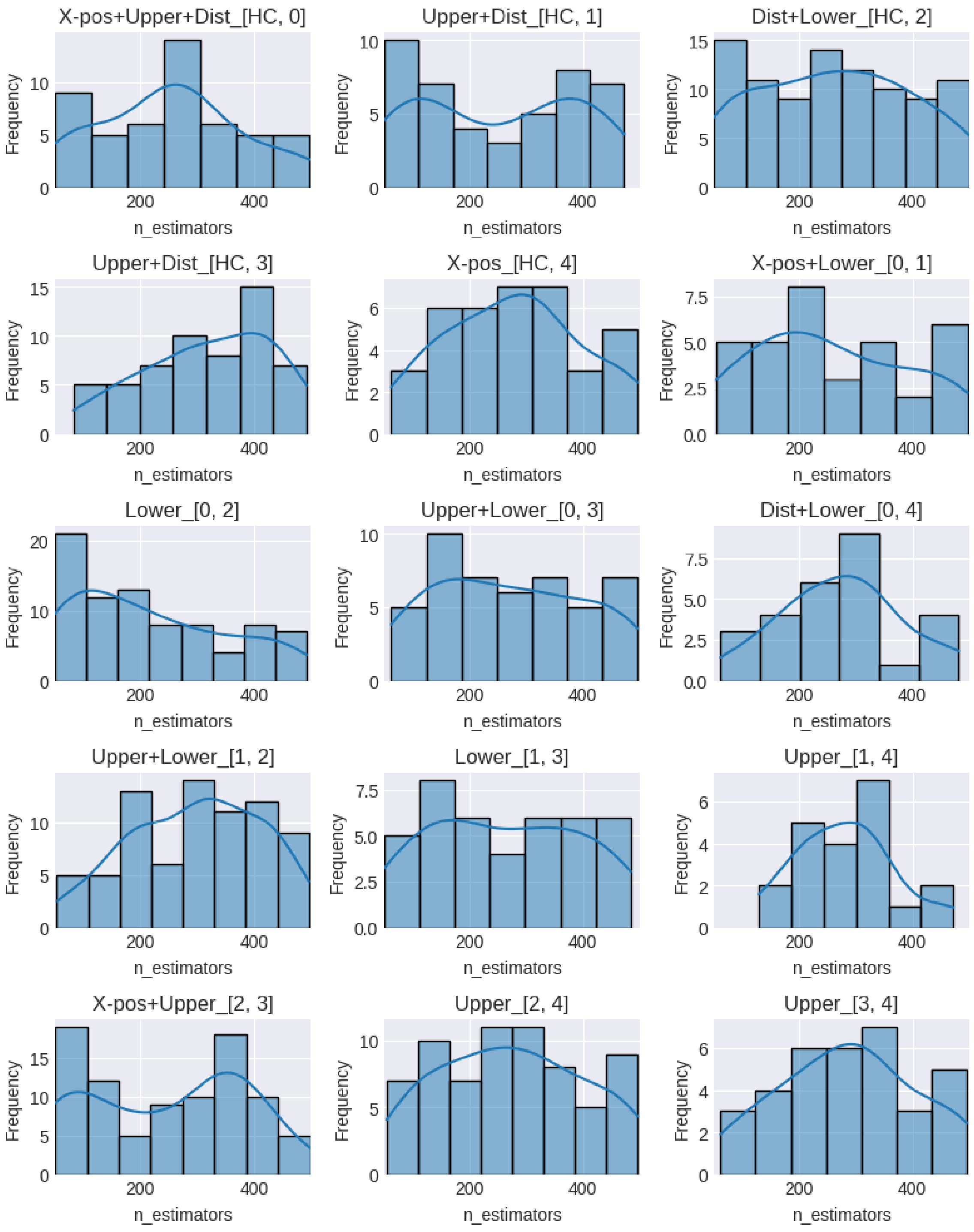

Supplementary Figure 20: **Histograms of n estimators hyperparameter during the leave-one-out cross-validation of the tsfresh+XGBoost classification experiment.**
Each plot presents the distribution of the n estimators hyperparameter in a certain binary classification scenario, indicated in the plot title alongside the time series (combination) utilized. For instance, the upper left plot shows the distribution of the hyperparameter for the binary classification trained and evaluated on the classes healthy controls (HC) and SARA gait score 0. The histograms allow insight into the distribution of favorable n_estimators values during the cross-validation of the best-performing regression model, namely tsfresh+XGBoost. Additionally, each histogram displays a kernel density estimate curve overlaid on top of the histogram bars. Details on the hyperparameter search spaces are given in Supplementary Table 3.

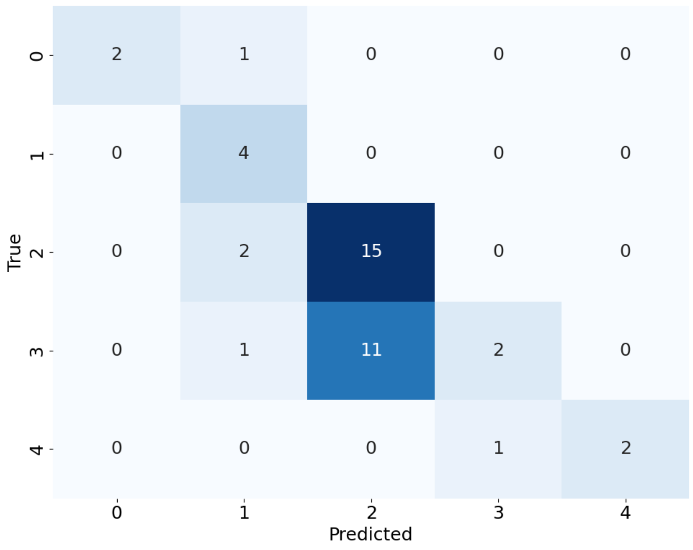

(a) tsfresh+XGBoost$_{H41}$

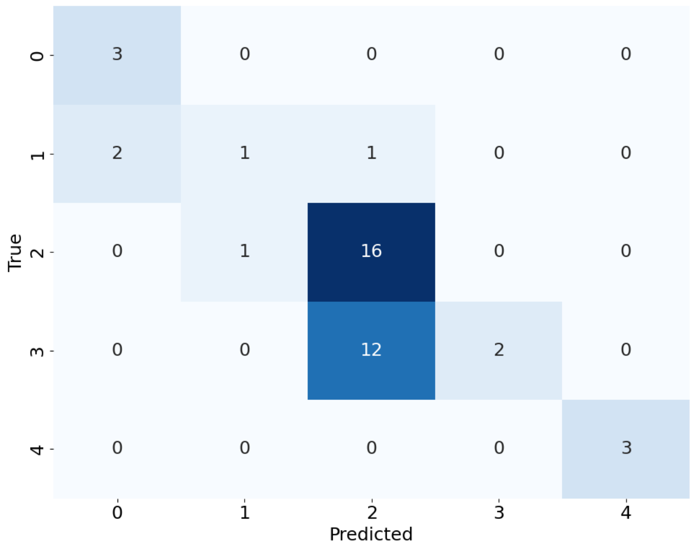

(b) Human baseline.

Supplementary Figure 21: **Full confusion matrices for the tsfresh+XGBoost$_{H41}$ and human baseline experiments predicting the SARA gait score on the ordinal scale** $[0, 1, 2, 3, 4]$**.**
All predictions are presented against the true values in a matrix with the predicted scores listed row-wise and the true values column-wise. A perfect result holds values solely on the main diagonal. Every instance not on the main diagonal is a misclassified sample. The tsfresh+XGBoost$_{H41}$ experiment is presented in the left figure (a) with the human baseline presented in the right figure (b).

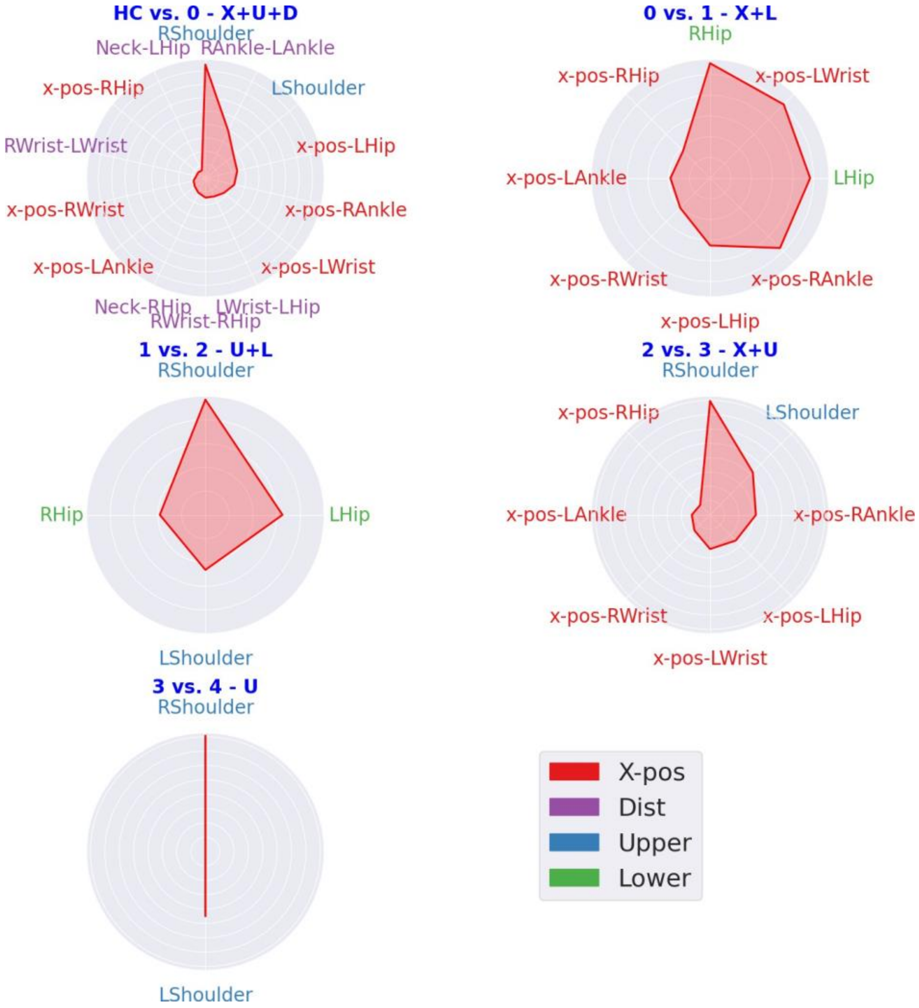

Supplementary Figure 22: **Results of the explainability analysis concerning the binary classification experiments illustrated in a radarplot with clockwise decreasing importance values beginning at 12 o'clock.**
Feature importance in direct neighbor comparisons in terms of SHAP values. Binary classifications of the neighboring classes: HC versus ataxia patients rated with a SARA gait score of 0; ataxia patients rated with a SARA gait score of 0 versus 1, etc.. Shap values are taken from the binary classification experiment for the model tsfresh+XGBoost.

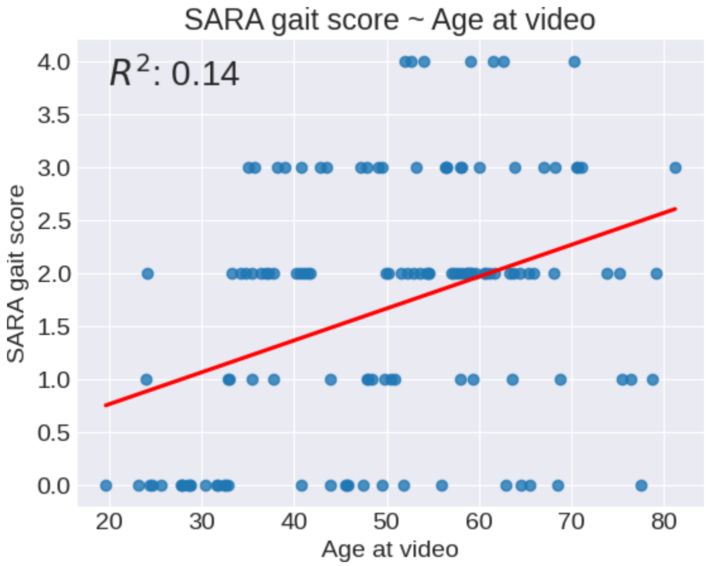

Supplementary Figure 23: **Scatter plot of the age at the assessment on the x-axis and the SARA gait score on the y-axis with a linear regression overlayed.**
Linear regression analysis of the age and the SARA gait score at a given assessment for the entire cohort of 159 participants, including controls. The age at the assessment predicts the respective SARA gait score with an R^2-score of 0.14.

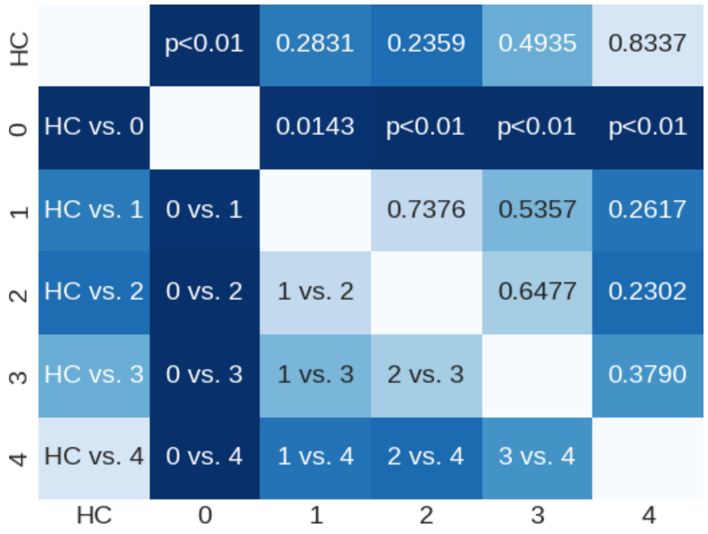

Supplementary Figure 24: **Test results of investigating age differences between sub-groups of the cohort defined by different SARA gait scores.**
Results of a statistical test (two-sided Mann-Whitney U-Test) comparing ages between certain SARA-gait-score sub-cohorts. The results are presented in a color-coded matrix indicating the respective p-values. Very significant results ($p<0.01$) are indicated as such, and the exact p-value is not reported in the figure. However, the exact p-values are: (HC, 0): 0.00047, (0, 2): 0.00014, (0, 3): 0.00050, (0, 4): 0.0037.
